# Unifying Gene Expression Measures from Multiple Platforms Using Factor Analysis

**DOI:** 10.1371/journal.pone.0017691

**Published:** 2011-03-11

**Authors:** Xin Victoria Wang, Roel G. W. Verhaak, Elizabeth Purdom, Paul T. Spellman, Terence P. Speed

**Affiliations:** 1 Department of Biostatistics and Computational Biology, Dana-Farber Cancer Institute, Boston, Massachusetts, United States of America; 2 Department of Biostatistics, Harvard School of Public Health, Boston, Massachusetts, United States of America; 3 Department of Bioinformatics and Computational Biology, MD Anderson Cancer Center, Houston, Texas, United States of America; 4 The Eli and Edythe L. Broad Institute of Massachusetts Institute of Technology and Harvard University, Cambridge, Massachusetts, United States of America; 5 Department of Statistics, University of California, Berkeley, California, United States of America; 6 Life Sciences Division, Lawrence Berkeley National Laboratory, Berkeley, California, United States of America; 7 Bioinformatics Division, Walter & Eliza Hall Institute of Medical Research, Parkville, Australia; University of Leuven, Belgium

## Abstract

In the Cancer Genome Atlas (TCGA) project, gene expression of the same set of samples is measured multiple times on different microarray platforms. There are two main advantages to combining these measurements. First, we have the opportunity to obtain a more precise and accurate estimate of expression levels than using the individual platforms alone. Second, the combined measure simplifies downstream analysis by eliminating the need to work with three sets of expression measures and to consolidate results from the three platforms.

We propose to use factor analysis (FA) to obtain a unified gene expression measure (UE) from multiple platforms. The UE is a weighted average of the three platforms, and is shown to perform well in terms of accuracy and precision. In addition, the FA model produces parameter estimates that allow the assessment of the model fit.

The R code is provided in File S2. Gene-level FA measurements for the TCGA data sets are available from http://tcga-data.nci.nih.gov/docs/publications/unified_expression/.

## Introduction

The Cancer Genome Atlas (TCGA) project [1], [2] aims to understand the molecular basis of cancer by characterizing different aspects of the cancer genome, including copy number, methylation, mutation and gene expression. In the first phase of the project, three microarray platforms are used to measure gene expression levels in glioblastoma multiforme (GBM), ovarian and lung squamous tumor samples: Affymetrix Human Genome HTS U133A Array (U133), Affymetrix GeneChip Human Exon 1.0 ST Array (Exon), and Agilent custom 244K Array (Agilent), giving us three sets of gene expression measures on all the samples.

This article addresses the problem of combining gene expression measures for the same set of samples from multiple platforms. A combined measure is desirable because 1) we get a more reliable estimate by using more information, and 2) downstream analysis can be carried out on a single set of expression measures rather than multiple. As shown in [3], different microarray platforms have different performances, therefore simply taking an average of the measurements from different platforms would not necessarily be the optimal solution.

Our method was used to generate the unified expression measures for TCGA glioblastoma multiforme (GBM) data, which was used in Verhaak et al [4]. Other than the limited description of our method there, we are not aware of any other work that addresses this particular problem. However, much work has been done to address the problem of combining expression data from different platforms, where different samples are measured by different platforms. For example, Warnat et al [5] proposed to use median rank scores and quantile discretization to integrate expression data from different studies for the purpose of supervised classification analysis. Scharpf et al [6] proposed a hierachical Bayesian model to integrate data from different studies and to identify differentially expressed genes between two conditions.

Here, we propose to use factor analysis to address this problem. The main advantage of using factor analysis to combine concomitant expression measures is that it gives each platform a weight depending on how well its measurements correlate with the rest of the platforms and therefore has the ability to down-weight a problematic platform. The underlying gene expression level is reflected in the measurements obtained from each of the platforms and is thought of as the latent variable in the factor analysis model. Factor analysis has previously been used in the single-platform setting as a summarization method for Affymetrix U133 platforms [7], and was shown to perform comparably with Robust Multichip Average (RMA) [8], which is a widely used summarization method in the single-platform setting.

Factor analysis can be applied in two ways. One is to first summarize the expression measures within each platform and apply factor analysis to the platform summaries. We call this gene-level FA. This method requires the gene of interest to be measured on at least three platforms simultaneously. The second method, probe-level FA, applies factor analysis directly to probe-level measurements across all platforms. This eliminates the requirement of having at least three platforms measure a given gene and can provide estimates for all genes that are measured on any of the platforms. Factor analysis as we do it requires more samples than platforms, and is best when the number of samples greatly exceeds the number of platforms. Our approach will not apply in a setting such as the MAQC project [9], where the number of samples and the number of platforms are very similar.

We have applied both gene-level and probe-level FA to integrate the expression measures from U133, Exon and Agilent arrays for TCGA GBM and ovarian tumor samples. We use expression levels measured from digital sequencing (DGE) experiments on a subset of these samples as our gold standard to evaluate the performance of our FA models, as digital sequencing has been shown to be highly reproducible and accurate in identifying differentially expressed genes [10], [11]. A simulation study has been carried out to further understand the performance of gene-level FA.

We compare the performances of gene-level and probe-level FAs to each of the three platforms alone, as well as gene-level and probe-level averages in terms of precision and accuracy. We find that gene-level FA and gene-level average are the top performers when evaluated by several criteria across all genes, followed by probe-level FA. Our simulation study further shows that gene-level FA has a clear advantage over gene-level average when two platforms are giving good expression measurements and the third is mis-behaving. This result is confirmed when we look at the subset of such genes in the TCGA ovarian data set.

## Methods

### Data

We have applied the proposed FA models to 246 GBM samples and 175 ovarian samples available through TCGA. This paper focuses on the ovarian data set where digital sequencing data is available for 31 of the 175 samples, which we use as a gold standard to evaluate our methods. Each sample is measured on three microarray platforms: U133, Exon and Agilent. Probes from the three platforms are mapped to a transcript database compiled from RefSeq and GenBank as described in [2], [4]. For the Affymetrix platforms, a minimum of five perfect matching probes is required to define a new, gene-centric probe set. A minimum of three perfect matching probes is required for the sparser Agilent array. The new gene-centric probe set definitions ensure independence from the manufacturer annotation and allow direct comparisons between the different platforms. Using the new definitions, 11,864 genes are represented on all three platforms and are included in our analysis. Gene summaries are obtained through the TCGA data portal (cancergenome.nih.gov) and are used for gene-level FA. Background-corrected and normalized probe-level data, generated from the raw data files available through the TCGA portal, are used for probe-level FA. Please refer to the TCGA Data Primer [12] for a detailed description of sample acquisition, processing and data generation.

### Proposed model

A thorough presentation of the factor analysis model can be found in [13]. Here we provide a brief description of the model when applied to generate the unified expression measure. Let 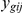 be the observed expression measure on the log scale for gene 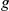 from sample 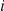 and platform 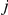, standardized to have mean 0 and variance 1 across all the samples within each platform. For the probe-level model, 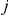 indexes probes both within and across all platforms. Let 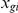 denote the unobserved underlying expression level we would like to estimate for gene 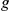 and sample 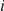. Since the factor analysis model is always applied gene by gene, we simplify notation by omitting the subscript 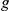 from now on. The factor model can be written as the following: 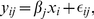
(1)where 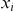 is called the common factor, and the error term 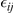 is called the specific or unique factor in factor analysis literature. If we consider 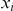, 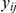 and 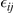 to be realizations of the random variable 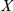, 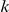-dimensional random vector 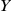, and 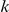-dimensional random vector 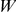 respectively, where 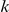 is the number platforms in gene-level FA, or the number of probes in probe-level FA, the model can be written as 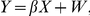
where 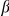 is a vector of length 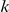, and the model is parameterized in the following way:
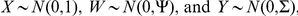
where 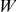 is independent of 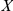, 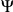 is 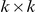 and diagonal, and 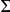 is 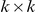 and has all 1's on the diagonal. Here 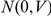 means multivariate normally distributed with mean 0 and variance-covariance matrix 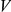, with the appropriate specialization if the dimension is 1. The following are readily derived: 
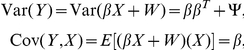



and
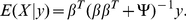
(2)


It turns out that the right hand side of (2) is equal to 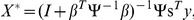
which is call the Thomson's factor score, also known as the regression factor score [14]. The Thomson's factor score inverts a matrix of smaller dimension than the conditional expectation in (2), and is used here to estimate the common factor score 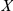.

In order to obtain unified expression measures for a given gene using Thomson's factor score, we need to estimate the parameters 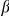 and 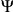. The log likelihood of 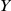 is 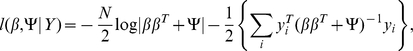



and the maximum likelihood estimates of the parameters 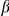 and 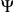 are obtained using an EM algorithm. Note that the likelihood is invariant to orthogonal transformations of 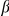, which is simply sign flipping in this case, since 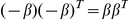.

Thomson's factor score says that the unified expression measure is a weighted average of the observed expression measures from the 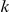 platforms, the vector 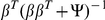 being the weights given to the platforms. Note that 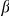 is the correlation between observed expression measure on different platforms and the unified expression measure we estimate, and that the weight given to a platform increases with the 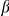 for that platform. The weights do not necessarily sum up to 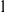, and can be negative in some cases.

We obtain the same unified expression measure even if the 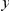
's are mean-centered but not standardized as the factor analysis model is scale invariant [14].

The factor analysis model is implemented using the R package 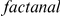.

## Results

We find that factor analysis gives unified expression measures that are highly correlated with well-performing platforms when a gene is expressed and has a reasonable dynamic range across samples. When a gene is expressed at background levels or has constant expression levels across samples, we cannot get a good estimate of the covariance matrix for that gene, and the factor analysis model will not produce helpful results. However, these are genes that are not of interest in most cases. By combining multiple platforms, discordant measures from one platform can be rescued by concordant measurements from the other two platforms. In these situations, the factor model gives a more reliable estimate than simply averaging measurements from all the platforms.

This section is organized as follows. We first describe the model fit for gene-level FA and probe-level FA by showing a few genes that are representative of the different situations, followed by a comparison of gene-level FA and probe-level FA. We then systematically evaluate the performance of our FA models in terms of precision (variance) and accuracy (bias) by first comparing them to five other gene expression summaries across all genes: gene-level averages of the three platforms, probe-level averages across all probes from the three platforms, and the three expression measures from the three platforms. The accuracy of gene-level FA and gene-level averages is then further compared using a simulation study, the results of which are then confirmed in the TCGA ovarian data set.

### Model fit

#### Gene-level model

**Figure 1:**
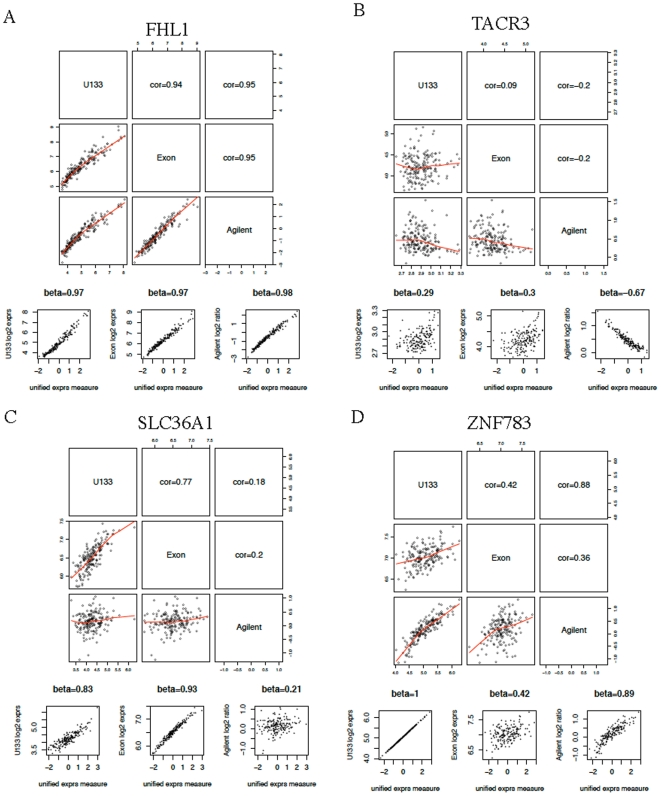
Four genes to illustrate gene-level FA: *FHL1* (A), *TACR3* (B), *SLC36A1* (C) and *ZNF783* (D). The top panels are pairs plots of the gene-level summaries of the three platforms. The bottom panels are scatter plots of gene-level summaries from each platform versus the unified expression values.

We illustrate the model fit in different situations using four genes, shown in Figure 1.

When pair-wise correlations between platforms are high (correlation
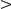
0.9, gene *FHL1*, Figure 1A), gene-level FA results in unified expression measures that are highly correlated with each of the platforms. Of the 11,864 genes present on all three platforms, 55% have all three pair-wise correlations 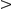 0.6, and 61% have all three 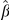
's 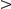 0.7. Recall that 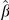
's are the correlations between each platform and the unified gene expression (Figures S1 and S2, Table S1).

**Figure 2:**
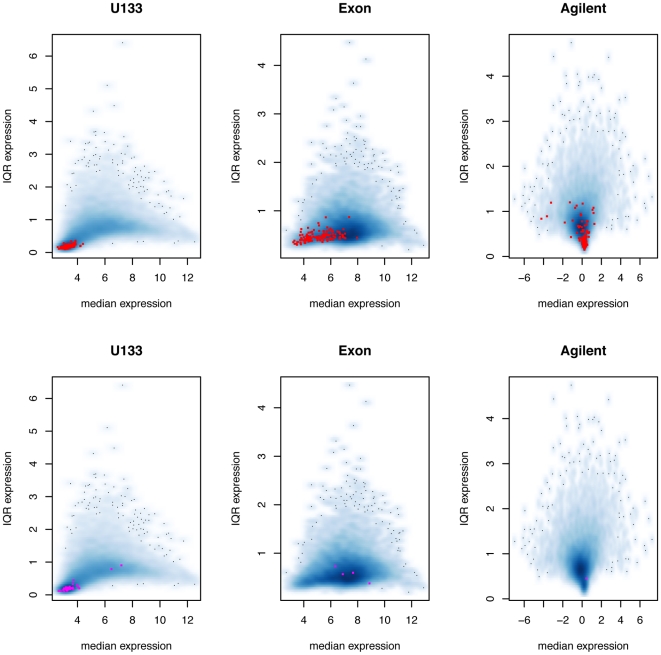
Medians and inter-quartile ranges (IQR's) of gene expression values. Each dot in these plots is a gene. The x-axis is the median expression value for a given gene over the 175 ovarian samples, and the y-axis is the IQR for that gene over the 175 samples. In the top row, 129 genes with all three 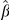
's 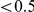 are highlighted in red. In the bottom row, 40 genes with one negative 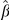 and two other 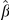
's 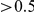 are highlighted in the platform where the 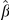 is negative.

When the three platforms do not correlate, e.g. due to low expression values or small dynamic ranges (e.g. gene *TACR3*, Figure 1B), the model is not very helpful. Although the unified expression measure correlates moderately with U133, it has a negative correlation of modest size with Agilent, which is an indication of poor model fit, and the results should be disregarded. A few hundred genes have small or even negative 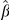
's (Tables S2 and S3), which are usually associated with platforms with low expression values or small dynamic ranges (Figure 2).

For 409 genes, two platforms correlate well with each other, but not the third platform (one correlation 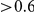 and two others 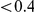). In all of these cases, the two concordant platforms are assigned high 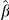 values and the platform not correlating with the rest gets a low 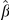 value (3). This may be related to mis-annotation or novel gene structures. An example is the gene *SLC36A1* (Figure 1C), where the model gives unified expression measures mostly based on U133 and Exon, which have good correlation. A similar pattern is seen in the GBM data set for this gene (Figure S4). DGE data also correlates better with U133 and Exon than with Agilent (Figure S5). Upon further inspection, we find the Agilent and U133 probes to target the 3′ UTR region of one of four possible transcripts of *SLC36A1* with U133 probes on the 3′ side of Agilent probes, while Exon probes target all four possible transcripts, according to Ensembl build hg19. It is possible that the U133 probes target a novel transcribed region that is part of the other three transcripts. There are 53 genes with one bad 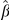 value (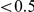) when the gene is expressed at levels generally considered to be above background (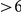 for U133 and Exon, 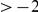 for Agilent) with a dynamic range of 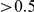 (as measured by the inter-quartile range, IQR) on all three platforms, which are likely due to annotation errors. For all of these cases, the low 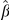 value is associated with the platform that is not concordant with the other two (Figure S6).

In the last example (gene ZNF783, Figure 1D), the exon array is at a reasonable expression level but with a relatively small dynamic range of 0.6 and does not correlate with measurements from U133 or Agilent. In this case, unified expression measure is largely the U133 measure. A situation such as this, where one of the 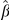 is near 1 is called a “Heywood case” in the factor analysis literature. This is due to the constraint of 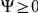. Heywood cases arise when the optimization procedure yields a solution with negative 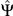 entries. There are 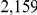 genes in the ovarian data set where Heywood cases occur. A large percentage of them are genes at low expression levels and with small variation across samples, but quite a few also occur when a gene is highly expressed or variable (Figure S7). In all but 9 such genes, the platform with the negative 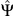 entry (and therefore a 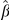 near 1) is one of the two platforms with the best pair-wise correlations. Therefore although undesirable, the estimates obtained in these cases are still likely to be reasonable, i.e. with best performing platforms assigned highest weights, but should be used with caution. There are 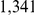 Heywood cases in the GBM data set, where there are 246 samples, consistent with the observation that they are less likely to occur with the increase of sample size [13].

We use the bootstrap [15] to estimate the standard errors of our 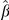. For most genes, the SE of 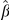 is less than 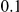 (Figure S8), meaning that the unified gene expression estimates have reasonable variances in most cases. Genes with large 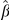 SE's (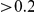) are usually expressed at low levels or have small dynamic ranges (Figure S9). Of the genes with large 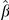 SE's and IQR's greater than 1 for exon and Agilent, only three have U133 median expression levels greater than 4: *RPS4Y1*, *COX6A2* and *RPL23AP13*, which all have reasons to have unstable 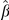 estimates: *RPS4Y1* has one very obvious outlier sample in U133; *COX6A2* has two obvious outlier samples in Agilent; *RPL23AP13* does not have any obvious outlier samples but its gene summaries on the three platforms do not have much correlation.

#### Probe-level model

Probe-level FA is applied to the 175 ovarian samples run on the same three platforms: U133, Exon, and Agilent for the 11,864 genes represented on all three platforms. Instead of applying the FA model on gene summaries from each of the platforms, we now apply the model directly to background-corrected and normalized probe measurements from all three platforms, i.e. 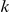 is now the sum of the number of probes on the three platforms, whereas in the gene-level model, 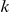 is 3, the number of platforms.

The three platforms have different numbers of probes interrogating a given gene. The exon array has the greatest number of probes, usually between 20 and 100. U133 has 11 probes per gene for the majority of the genes. Agilent has about 2 to 8 unique probes per gene, and each probe is usually replicated two to three times. So for a given gene, there are usually a total of 60 to 200 probes from the three platforms (Figure S10).

Two genes used as examples in the gene-level FA discussion are shown here to illustrate the performance of probe-level FA.

**Figure 3:**
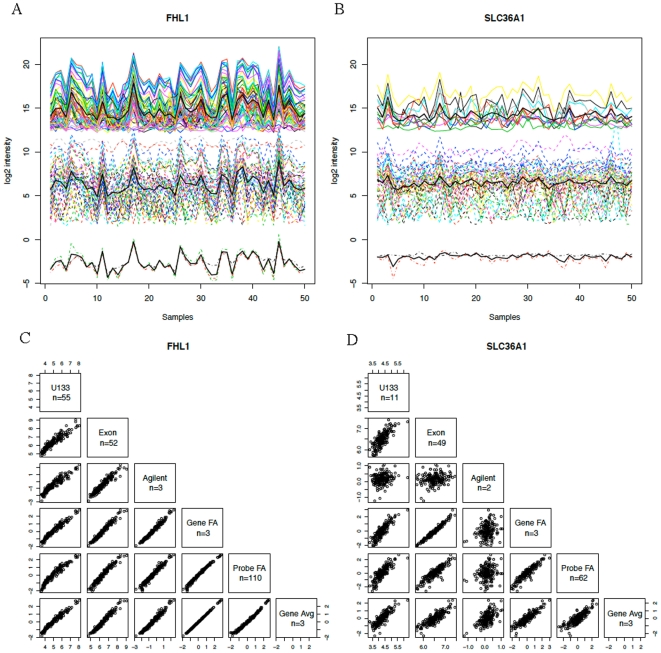
Probe-level factor analysis. A, B: Log2 probe intensities of *FHL1* and *SLC36A1*. Solid lines are U133 probes, dashed lines are Exon probes, and dot-dashed lines are Agilent probes. The thicker solid black lines are gene summaries for U133, Exon and Agilent. For ease of visualization, a constant of 10 and −2 are added to U133 and Agilent, respectively. Only the first 50 samples are used. C, D: Pairs plots of different gene summaries. From top to bottom: U133, Exon, Agilent, gene-level FA, probe-level FA and gene-level average.

In the case of highly expressed gene *FHL1* (Figure 3A), most probes track each other very well with a lot of variation across samples. Some probes on the U133 and exon arrays do not work as well (Figure S11). The exon array has the lowest within-platform probe correlations, possibly due to alternative splicing, and results in lower 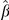 estimates (Figure S12). Outlying probes on U133 are also assigned lower 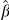 estimates. With problematic probes down-weighted, it is not surprising to see in Figure 3C that all reasonable methods give highly correlated estimates.

Of course, not all genes perform as well as *FHL1*. In fact, if all genes looked as perfect as *FHL1*, a simple average would suffice and there would be no need for a method that down-weights bad platforms or probes. For the *SLC36A1* gene, the Agilent probes do not respond the same way as the two Affymetrix platforms (Figure 3B, Figure S13). U133 and Exon arrays have higher pairwise correlations and get higher 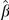 values (Figure S14), resulting in the factor analysis model basing its estimates mostly on the two Affymetrix platforms (Figure 3D). We can also see from the same figure that the average of the platform gene-level summaries is influenced by the under-performing Agilent probes, which is what we would like to avoid with the FA model.

**Figure 4:**
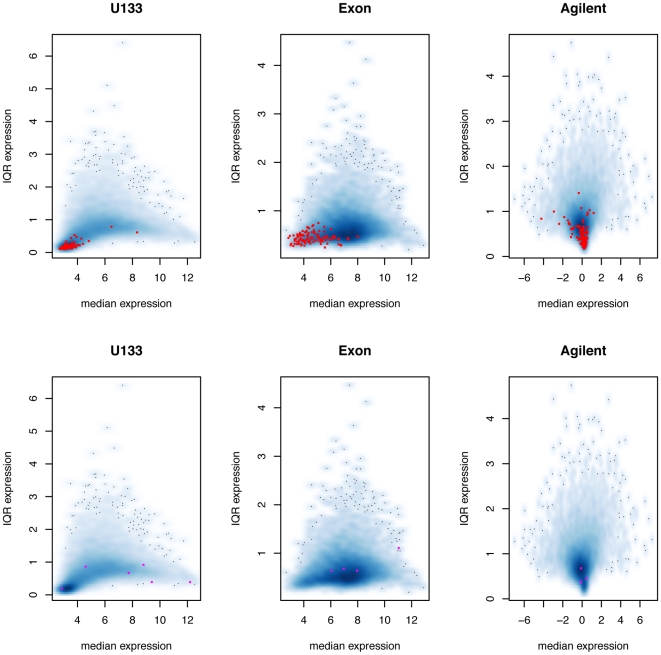
Medians and inter-quartile ranges (IQR's) of gene expression values. Each dot in these plots is a gene. The x-axis is the median expression value for a given gene over the 175 ovarian samples, and the y-axis is the IQR for that gene over the 175 samples. For a given gene, a median 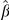 is calculated for each platform. In the top row, 114 genes with all three median 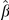
's 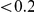 are highlighted in red. In the bottom row, 14 genes with one negative median 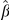 and two other median 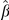
's 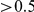 are highlighted in the platform where the median 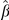 is negative.

In the probe-level model, the unified gene expression measure is a weighted average of the probe measurements, and each probe has a 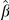 value associated with it. The larger the 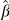, the larger the contribution from that probe to the unified measure. Figure 4 is the probe-level equivalent of Figure 2 in the gene-level model. For each platform, we highlight genes with low median probe 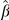 values, and as in the gene-level model, they occur when the genes have low expression values or small dynamic ranges. There are 69 genes in which Heywood cases occur in the probe-level model fit, many fewer than in the gene-level model fit.

#### Comparing gene-level and probe-level FA

Gene-level FA and probe-level FA have their own advantages. Gene-level FA combines only three measurements into one, and takes minutes to compute. Its disadvantage is that it can only work for genes that are present on all three platforms. Probe-level FA eliminates the requirement for a gene to be present on all three platforms, but takes hours to compute, and for some genes, fails to converge (149 in the GBM dataset and 396 in the Ovarian dataset). Despite these differences, 75% of the genes have gene-level and probe-level FA correlations greater than 0.8 (Figure S15). There are 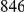 genes where the correlation between gene-level and probe-level FA estimates is less than 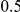. The majority of these genes have either low expression levels or small dynamic ranges (Figure S16). After these are removed (genes with U133 median 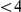, U133 IQR 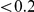, Exon median 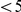, Exon IQR 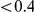, Agilent median 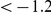, and Agilent IQR 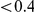), there are 76 genes left with probe-level and gene-level FA correlations less than 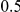. Among these, gene-level FA for 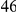 genes are Heywood cases, and therefore are not useful. For the remaining 30 genes, all but four are such that U133 and Agilent gene-level summaries correlate but not exon gene-level summaries. Gene-level FA for these genes are mostly based on Agilent and U133 gene-level summaries, as expected. However, probe-level FA estimates correlate better with Exon gene summaries instead, which is the result of the many more probes present on the Exon array than the other two platforms. When measurements from the majority of these probes do not agree with the other two platforms, the gene summaries do not agree with the other two platforms either and get down-weighted in the gene-level FA. But in probe-level FA, these probes become the majority and are the driving forces of the probe-level FA estimates, which then do not agree with gene summaries of the other two platforms. An example is the *CCDC85B* gene (Figure S17), where gene-level FA is more accurate than probe-level FA according to DGE data. For the aforementioned 30 genes, the majority have better correlations between gene-level FA and DGE than between probe-level FA and DGE (Figure S18). Based on these observations, gene-level FA seems to be a more reliable method than the probe-level FA when the number of Exon array probes is large.

### Evaluation

To combine gene expression measures from several platforms, the naive method would be to take the mean of the standardized gene-level summaries of each platform. An alternative would be to take the mean of the probe-level data from all platforms, or use gene-level summaries from one of the platforms. Therefore we have seven gene expression summaries: gene-level averages, probe-level averages, RMA summaries of U133 arrays, RMA summaries of Exon arrays, gene summaries of Agilent arrays, gene-level FA and probe-level FA. We now evaluate these seven gene expression summaries in terms of precision (variance) and accuracy (bias). For precision, we use the nine sets of replicate samples in our data and compare the variability of the seven gene expression summaries. For accuracy, we use digital sequencing data on 31 of the ovarian tumor samples as our gold standard. Note that notations used in this section is independent of those in the methods section.

#### Comparison of precision

**Figure 5:**
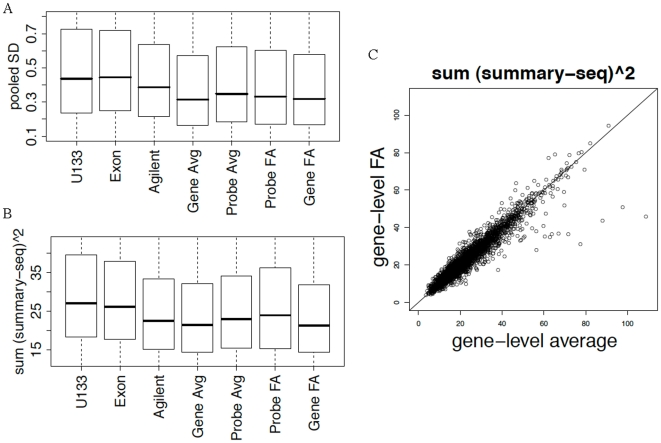
Comparisons of precision and accuracy. A: Pooled standard deviations of standardized gene expression measures. The medians are 0.44, 0.44, 0.39, 0.31, 0.35, 0.33, 0.32 from left to right. B: Sum of squared differences between array summaries and tag profiling. The medians are 27.0, 26.1, 22.5, 21.4, 22.9, 23.9 and 21.3 from left to right. C: Sum of squared differences between gene-level averages and tag profiling, and between gene-level factor analysis and DGE, for genes with exactly two 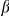
's 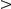 0.8.

There are 9 sets of replicate samples: 06-0137 (2), 06-0138 (2), 06-0145 (4), 06-0154 (2), 06-0156 (3), 06-0168 (2), 06-0176 (2), 06-0208 (2) and 06-0211 (2). For a given gene, let 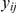 denote the standardized gene expression summary of the 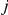 th replicate of sample 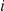. The pooled variance for each gene expression summary is 
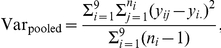
where 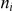 is the number of replicates for sample 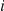. We standardize each gene expression summary to have mean 0 and standard deviation 1 for each gene across samples so that they are comparable. Figure 5A shows that gene-level FA and gene-level averages have the smallest pooled standard deviations, and that they give estimates that are the least variable.

#### Comparison of accuracy

Next-generation sequencing is emerging as an attractive alternative to microarrays for measuring gene expression levels. Instead of relying on hybridization of predetermined probes to target transcripts, the new sequencing technologies count the number of reads mapped to genes of interest, and thus eliminate issues with cross hybridization and background signals. Studies find that gene expression measured from next- generation sequencing is highly replicable and that its performance in identifying differentially expressed genes is highly concordant with qRT-PCR [10], [11]. Gene expression of 31 of the TCGA ovarian samples is measured using Illumina Tag Profiling, also known as Digital Gene Expression Tag Profiling (DGE) [16], a next generation sequencing technology based on serial analysis of gene expression (SAGE) [17], [18]. DGE has been shown to have major improvements in robustness, resolution and inter-lab reproducibility over microarray platforms ([19]). Therefore we use DGE data as a reference and compare the performance of the unified expression measures to that of the three platforms alone and simple averages.

We use median-normalized Illumina DGE gene count data for 31 of the ovarian tumor samples as our reference for expression levels. Processing of the DGE data is described in File S1.

#### Similarities between array summaries and DGE data

As a first step to assess how well array gene expression summaries resemble that of DGE data, we compute the sum of squared differences between standardized array summaries and standardized log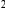 DGE data for each gene. For a given array platform, let 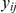 denote the standardized log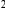 expression measure for gene 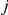 from sample 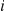, and let 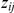 denote the standardized log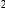 counts for gene 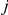 from sample 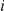 of the median-normalized counts. The sum of squared differences between an array platform and DGE for gene 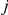 is 
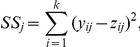



where 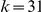 is the number of samples. Figure 5B shows the 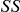 for the seven array summaries and we see that gene-level FA has the smallest median 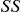 and gives expression measures that are closest to DGE.

#### Comparison of estimated fold changes

**Figure 6:**
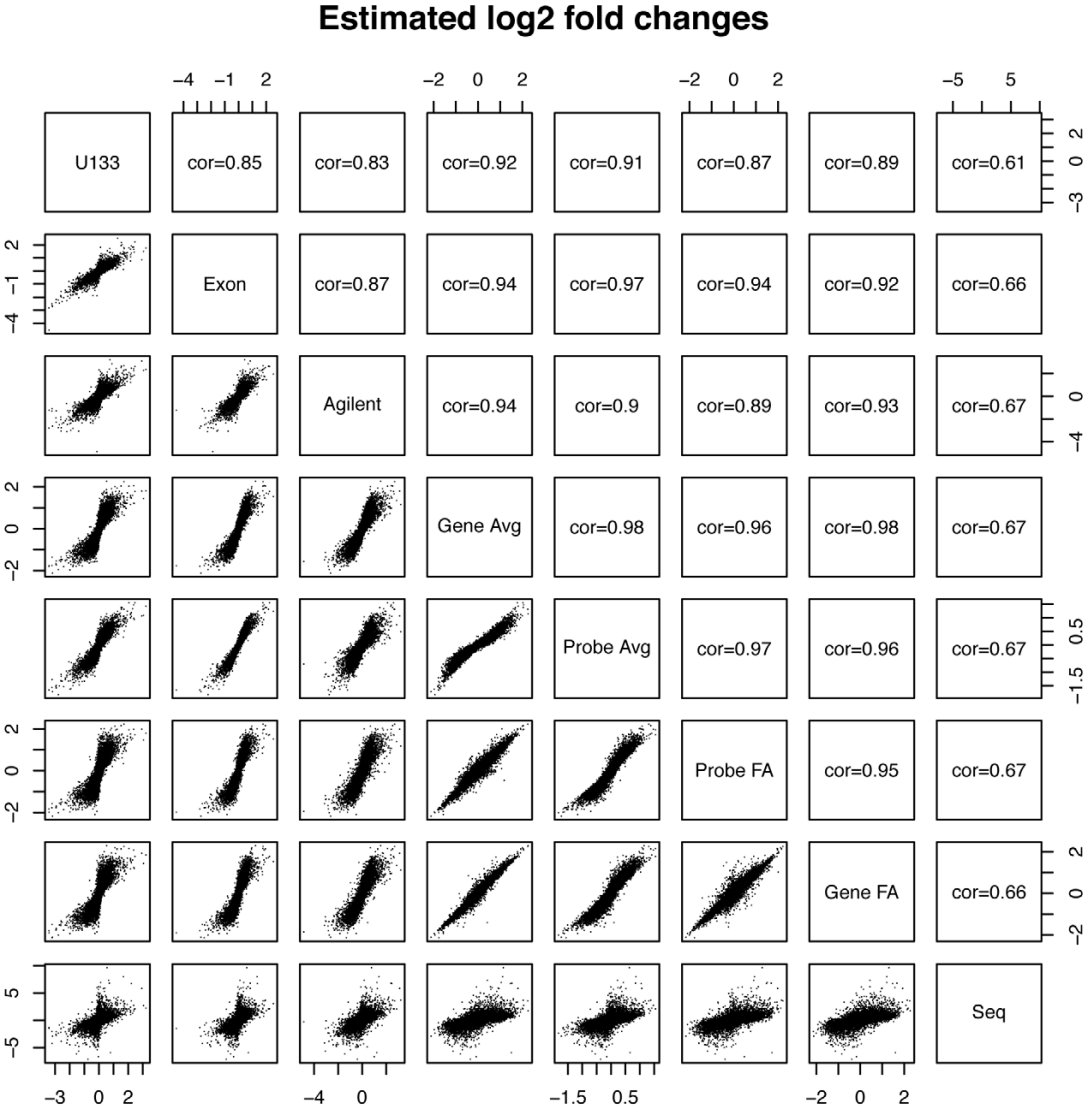
Pairs plot of estimated log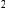 fold changes computed from different gene expression value estimates: U133 gene-level summaries, Exon array gene-level summaries, Agilent gene-level summaries, gene-level averages across the three platforms, probe-level averages across the three platforms, probe-level factor analysis estimates, gene-level factor analysis estimates, and digital sequencing gene-level summaries. Spearman correlations are shown.

Instead of quantifying absolute gene expression levels, we often use microarrays to compare the expression levels of two groups by estimating a fold change for each gene. Here we obtain two distinct groups of sizes 7 and 24 from the 31 samples by consensus clustering [20], denoted by 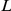 and 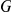 respectively (Figure S19), representing possible subclasses of ovarian cancer. The log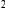 fold change from DGE for gene 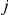 is estimated to be 
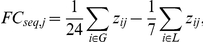



where 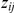 is the log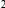 counts for gene 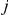 from sample 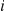 of the median-normalized counts. The log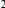 fold changes for array summaries are computed in the same way by replacing 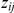 with log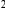 gene expression measurements 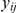 for each of the seven gene summaries obtained from array data. DGE produces fold changes that do not correlate as well as correlations among the array summaries (Figure 6), which is not very surprising as the two technologies are fundamentally different. The different gene summaries give fold change estimates that have similar correlations with DGE.

#### Identifying differentially expressed genes

Poisson regression models have been shown to work well across technical strata for digital sequencing data [10], [11]. However, we find significant departures from the Poisson distribution when examining qq-plots of 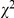 goodness-of-fit statistics (Figure S20), possibly due to biological variation among the samples.

**Figure 7:**
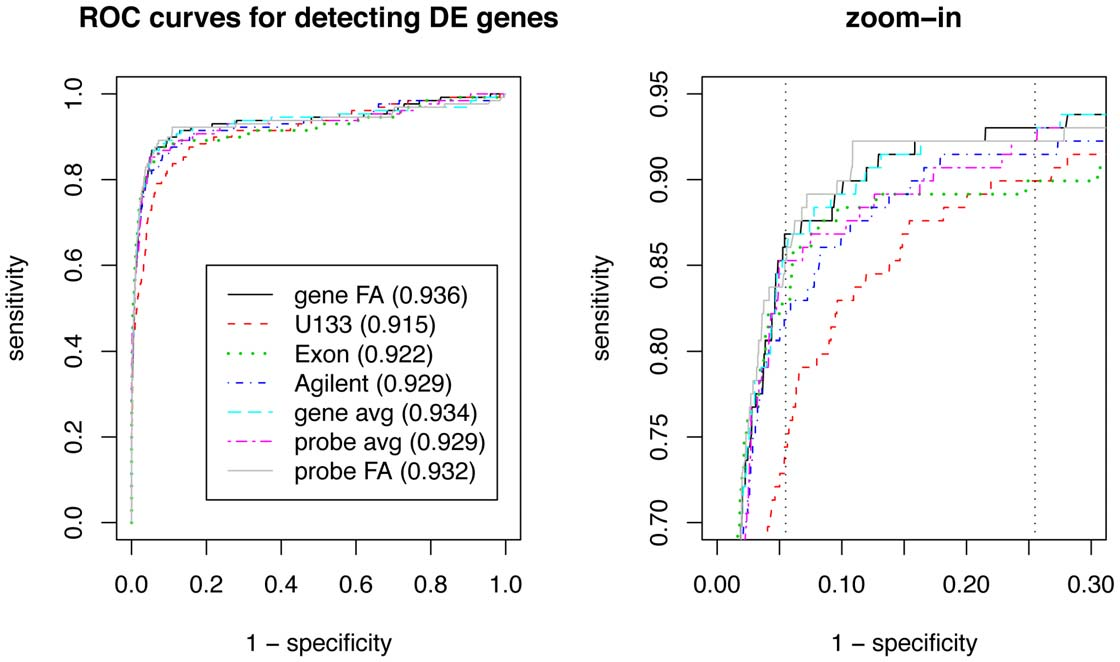
ROC curves constructed for the seven gene summaries. Genes with adjusted p-values 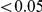 for DGE are considered true positives and genes with adjusted p-values 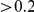 are considered true negatives. The area under the curves are indicated next to the gene summary methods in the legend.

We would like to test whether the log fold change between the two groups specified in section is significantly different from 0. Rather than relying on methods based on the Poisson distribution, we use *limma* [21], [22] to perform a two-sample comparison on the log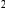 count data from DGE, and adjust for multiple testing using Benjamini and Hochberg [23]. We find 132 genes with adjusted p-values 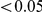 between the two groups from DGE data and regard them as true positives. There are 10,057 genes with adjusted p-values 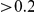, and are thought of as true negatives that do not display differential expression between the two groups. ROC curves constructed from these genes show that gene-level FA, probe-level FA, and gene-level averages perform the best in identifying differentially expressed gene, and that they are the most sensitive at specificities between 0.75 and 0.94 (Figure 7).

#### Summary of accuracy of gene expression measures

We use DGE data on 31 of the ovarian tumor samples to assess the accuracy of gene expression measurements from three microarray platforms, their simple averages, and the proposed unified expression measures. We find that the gene-level FA is the closest to the expression measures generated from DGE when evaluated by the sum of squared differences between standardized array summaries and standardized log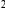 median-normalized count data from DGE, although gene-level averages of the three platforms perform almost as well. The seven gene summaries generate fold change estimates that have similar correlations with those generated by DGE. Gene-level FA, probe-level FAs and gene-level averages perform the best in identifying differentially expressed genes when genes with adjusted p-values 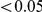 and 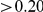 from DGE are used as true positives and negatives, respectively. With these findings, we feel confident that the unified expression measures produced from gene-level and probe-level FAs perform better than probe-level averages, and at least as well as simple gene-level averages in terms of accuracy.

### Further comparison of gene-level FA and gene-level averages

When looking across all the genes common to all three platforms, it appears that gene-level FA and gene-level average perform comparably. We perform a simulation study to evaluate their behaviors more systematically. Based on the results of this simulation study, we identify a subset of genes for which gene-level FA shows an advantage over gene-level average in TCGA ovarian data set.

#### The simulation procedure

We simulate gene expression data for 200 samples according to the FA model described in equation (1) as follows:

1. The true gene expression levels 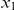, 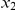, ..,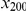 for a given gene of samples 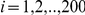 are simulated from 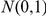;
2. For a given configuration of 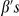, generate 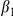, 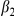 and 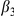 from uniform distributions of corresponding ranges. Then 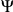 is determined since 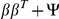 has all 1's on the diagonal;
3. Generate 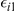, 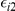 and 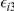 for 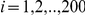 from 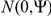;
4. Determine 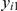, 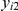 and 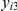 from 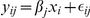 for 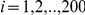;
5. Get estimates of 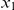, 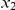, .., 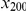 from 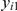, 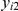 and 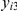, 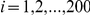, using gene-level FA and gene-level averages.
6. Repeat 200 times for each configuration of 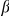.

Table 1 contains the different 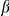 values we chose to simulate different situations that can arise in real data.

**Table 1:** Configurations of 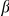
's used in simulations.

	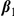 min	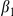 max	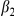 min	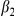 max	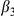 min	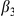 max
1	0.90	0.98	0.90	0.98	0.00	0.30
2	0.80	0.90	0.80	0.90	0.00	0.30
3	0.70	0.80	0.70	0.80	0.00	0.30
4	0.60	0.70	0.60	0.70	0.00	0.30
5	0.80	0.98	0.80	0.98	0.30	0.50
6	0.70	0.80	0.70	0.80	0.30	0.50
7	0.60	0.70	0.60	0.70	0.30	0.50
8	0.70	0.98	0.70	0.98	0.70	0.98
9	0.50	0.70	0.50	0.70	0.50	0.70
10	0.30	0.50	0.30	0.50	0.30	0.50
11	0.00	0.30	0.00	0.30	0.00	0.30
12	0.80	0.98	0.50	0.70	0.00	0.30

Two hundred genes are simulated for each configuration of 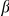. For a given range, 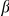
's are simulated from the uniform distribution with that range.

#### Simulation results

**Figure 8:**
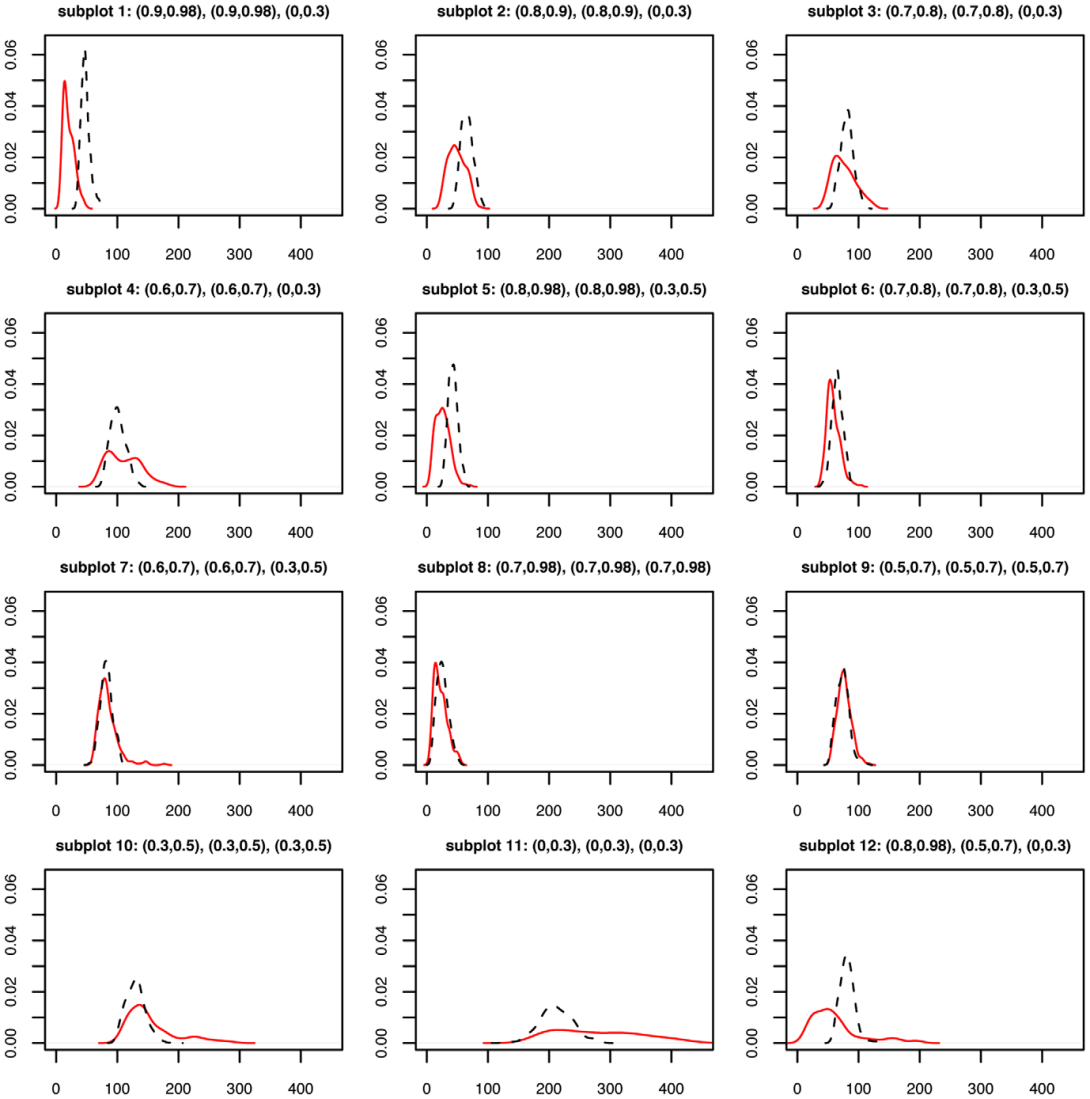
Simulation study. Solid line: density of sum of squared differences (x-axis) between the true expression level and gene-FA estimates. Dashed line: density of sum of squared differences between the true expression level and averages of the three platforms. Two hundred data sets are simulated from each 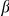 configuration. The ranges of the three 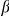 values are indicated at the top of each subplot.

Figure 8 shows the difference between estimated gene expression levels and true gene expression levels. Gene expression levels are estimated using gene-level FA and gene-level averages. The difference is represented by the sum of squared differences across the 200 simulated datasets for each 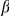 configuration.

We see that gene-level FA has a clear advantage over the average when two of the 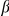
's are above 0.8 and the third 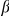 is below 0.5 (subplots 1, 2, 5). If the two best 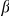
's are between 0.6 and 0.8 while the third 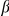 is below 0.3 (subplots 3 and 4), gene-level FA is sometimes better and sometimes worse than the average. If the third 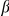 improves to between 0.3 and 0.5 (subplots 6 and 7), gene-level FA and the average become more similar to each other. The two estimates are comparable when all three 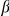
's are high (subplot 8 and 9). When one 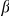 is high, one is low and the third is in between (subplot 12), gene-level FA does better most of the time, but worse than the average on occasions. Gene-level FA does worse when all three 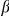
's are below 0.5 (subplots 10 and 11).

In general, the average is less sensitive than FA. The FA takes advantage of the two good platforms when it is clear that the third platform should be down-weighted, but can also produce worse estimates than the average when the situation is less clear.

#### Ovarian data

With more insight from our simulation study, we now look at a subset of genes where two of the array platforms have better than 0.5 correlations with DGE and the third platform has a correlation of 0.4 or less with DGE. There are 892 genes that fall into this category. We find that the gene-level FA has an advantage over the average in terms of distances to the expression level measured by DGE for this subset of genes, while they remain comparable in precision, fold-change and identification of DE genes (data not shown).

**Figure 9:**
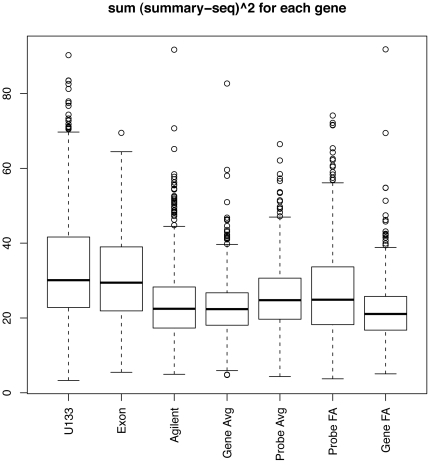
Sum of squared differences between array summaries and digital sequencing measures for the 892 genes that have two array platforms with 0.5 and above correlations with digital sequencing and the third array platform having a 0.4 and lower correlation with digital sequencing. The medians from left to right: 30.1, 29.4, 22.5, 22.4, 24.8, 24.9 and 21.1.

Figure 9 shows the sum of squared differences between different summaries and DGE. Gene-level FA performs the best with a median of 21.1, followed by gene-level average, with a median of 22.4. The difference between the two widens if we restrict to genes with two array and DGE correlations greater than 0.6 or the third array platform having a correlation of 0.3 or worse with DGE.

## Discussion

We propose to use the factor analysis model to produce a unified gene expression measure from three different microarray platforms (U133, Exon and Agilent) when the same set of samples is measured on each. Factor analysis is a natural method to use since it takes the covariance structure of the three platforms into account and estimates the unobserved variable as a weighted average of the three observed variables. This estimate is linear in the observed variables, albeit one with estimated coefficients.

The main advantage of the proposed factor analysis model is that it down-weights problematic platforms and therefore gives better estimates than a simple average. This is shown clearly in simulations (subplots 1 to 6). With real data, we do not expect the factor analysis model to show a drastic improvement over the simple average simply because the fraction of genes where one platform is misbehaving is small. However, when this does happen, the gene-level FA produces estimates that are more accurate than the simple average (Figures 5C). The other advantage of our method is that it produces parameter estimates (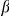 and 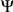) that can be used to evaluate the model fit and to filter out genes whose measurements are deemed unreliable, which was done for Verhaak et al [4].

The unified expression measures generated from the FA model was successfully used in a recent analysis of TCGA GBM expression profiles [4]. In this analysis, four robust unsupervised clusters were identified, which were associated with distinct genomic abnormalities in *IDH1*, *EGFR*, *PDGFRA* and *NF1*. Interestingly, the classifier cross-validation errors were reduced when gene-level FA expression levels were used, compared to training the classifier models on data from any of the three separate platforms. Reduction in cross-validation error has been shown to be a property of less noisy data [24].

TCGA is expected to produce expression profiles on three platforms on a total of 1,500 tumor samples of GBM, ovarian serous adenocarcinoma and lung squamous carcinoma, to which FA will be applied to produce unified expression estimates and will be made available at http://tcga-data.nci.nih.gov/docs/publications/unified_expression/. The factor analysis model may be further improved to produce good estimates when the correlation matrix is poorly estimated, which usually occurs for genes with a small dynamic range. An approach that allows us to borrow information from genes with well-estimated correlation matrices could improve the estimates for such genes.

## Supporting Information

Figure S1 Correlation of gene-level summaries among the three platforms per gene.
(TIF)

Figure S2Estimated 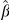 values of the three platforms per gene.(TIF)

Figure S3Estimated 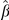 values for genes where one of the platforms does not correlate with the other two. These are genes where one pairwise correlation between two platforms is 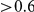 and two others are 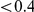. Gene counts are given in parenthesis in panel titles.(TIF)

Figure S4 GBM data. Gene SLC36A1: Gene-level summaries.
(TIF)

Figure S5 Gene SLC36A1: Gene-level summaries.
(TIF)

Figure S6Correlations for 53 genes with one bad 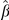 value (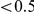) when the gene is expressed at a reasonable level (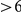 for U133 and Exon, 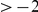 for Agilent) with a reasonable dynamic range (IQR 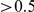) on all three platforms, which are likely due to annotation errors. The low 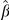 value is associated with the platform that is not concordant with the other two.(TIF)

Figure S7 Heywood cases. Red dots are genes in which Heywood cases occur.
(TIF)

Figure S8Bootstrap SE of 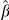.(TIF)

Figure S9Genes with large bootstrap SE of 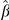. Red dots are genes with bootstrap SE of 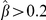.(TIF)

Figure S10 Number of probes per gene on each platform. Figures reflect the number of unique probes on the Agilent array, each usually duplicated 2 to 3 times.
(TIF)

Figure S11Pairwise probe correlations for gene FHL1 stratified into three within-platform groups and three between-platform groups. FHL1 has 55 U133 probes, 52 exon probes, and 3 Agilent probes. Therefore there are 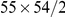 correlations within U133, 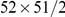 correlations within exon, 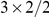 correlations within Agilent, 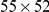 correlations between U133 and exon, 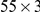 correlations between U133 and Agilent, and 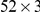 correlations between exon and Agilent.(TIF)

Figure S12Gene FHL1: probe-level 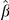 values stratified by platform.(TIF)

Figure S13Pairwise probe correlations for gene SLC36A1 stratified into three within-platform groups and three between-platform groups. SLC36A1 has 11 U133 probes, 49 exon probes, and 2 Agilent probes. Therefore there are 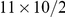 correlations within U133, 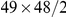 correlations within exon, 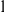 correlation within Agilent, 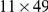 correlations between U133 and exon, 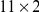 correlations between U133 and Agilent, and 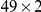 correlations between exon and Agilent.(TIF)

Figure S14Gene SLC36A1: probe-level 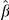 values stratified by platform.(TIF)

Figure S15 Histogram of 11,864 correlations between matching gene-level and probe-level gene expression estimates.
(TIF)

Figure S16Genes with low correlation between probe-level FA and gene-level FA. Red dots are genes whose correlation between probe-level FA and gene-level FA estimates are below 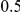.(TIF)

Figure S17 Gene CCD85B. Probe-level FA strongly influenced by Exon array because of its large number of probes.
(TIF)

Figure S18 Correlations between gene-level FA and DGE, and correlations between probe-level FA and DGE for 30 genes with low probe-level and gene-level FA correlations. These genes have reasonable expression levels and dynamic ranges, and are not Heywood cases.
(TIF)

Figure S19 Consensus clustering of the 31 ovarian samples. Samples 1 to 7 form cluster 1 (C1) and samples 8 to 31 form cluster 2 (C2).
(TIF)

Figure S20QQ-plot of 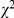 goodness-of-fit statistics for 24 samples from patients in cluster 2. Under the assumption of homogeneous Poisson rate for the gene counts, the goodness-of-fit statistics is 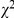 with 23 degrees of freedom. We see significant deviations from homogeneous Poisson distribution.(TIF)

Table S1Number of genes with varying 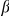 cutoffs for all three platforms.(PDF)

Table S2Percentage of small 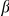
's.(PDF)

Table S3Number of genes with varying 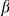 cutoffs for all three platforms.(PDF)

File S1 File describing the processing of DGE data.
(PDF)

File S2 R code.
(TXT)
