# Supplementary figures and images for: Unifying Gene Expression Measures from Multiple Platforms Using Factor Analysis
Source: PLoS One. 2011 Mar 11;6(3):e17691. doi: 10.1371/journal.pone.0017691 (PMC3059153; doi:10.1371/journal.pone.0017691)

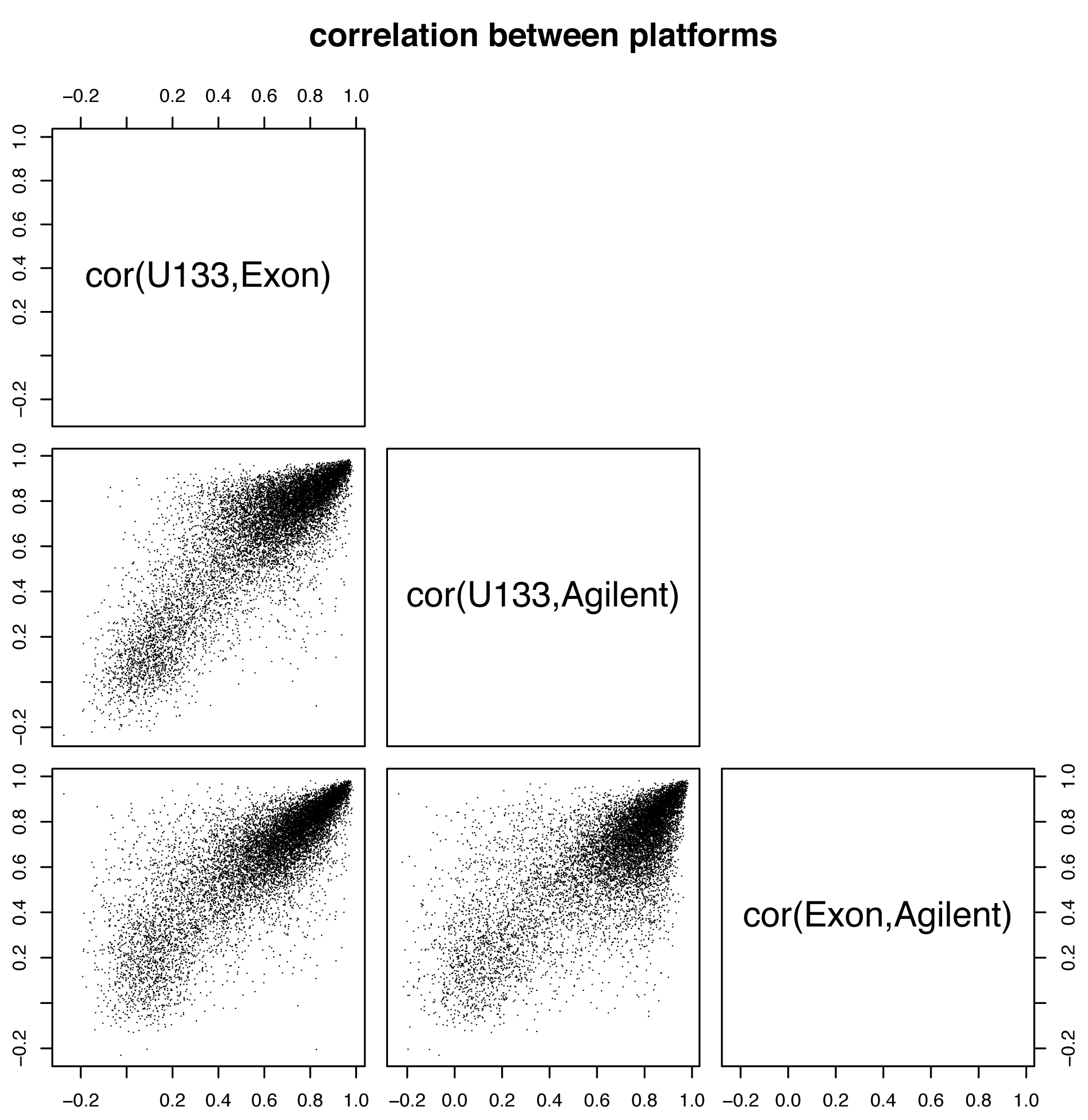

Supplement: Figure S1 — Correlation of gene-level summaries among the three platforms per gene. (TIF) [file pone.0017691.s001.tif]

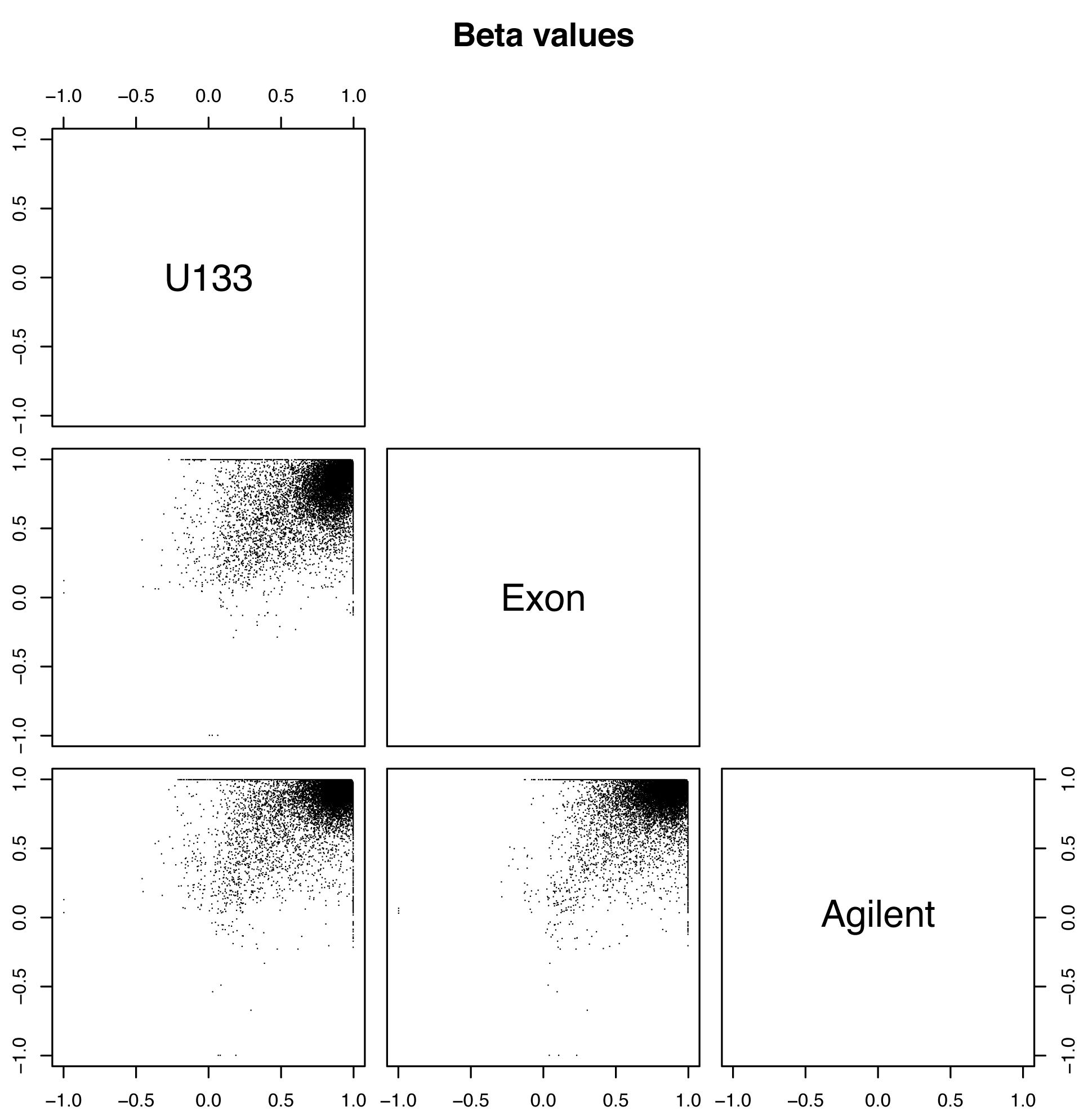

Supplement: Figure S2 — Estimated values of the three platforms per gene. (TIF) [file pone.0017691.s002.tif]

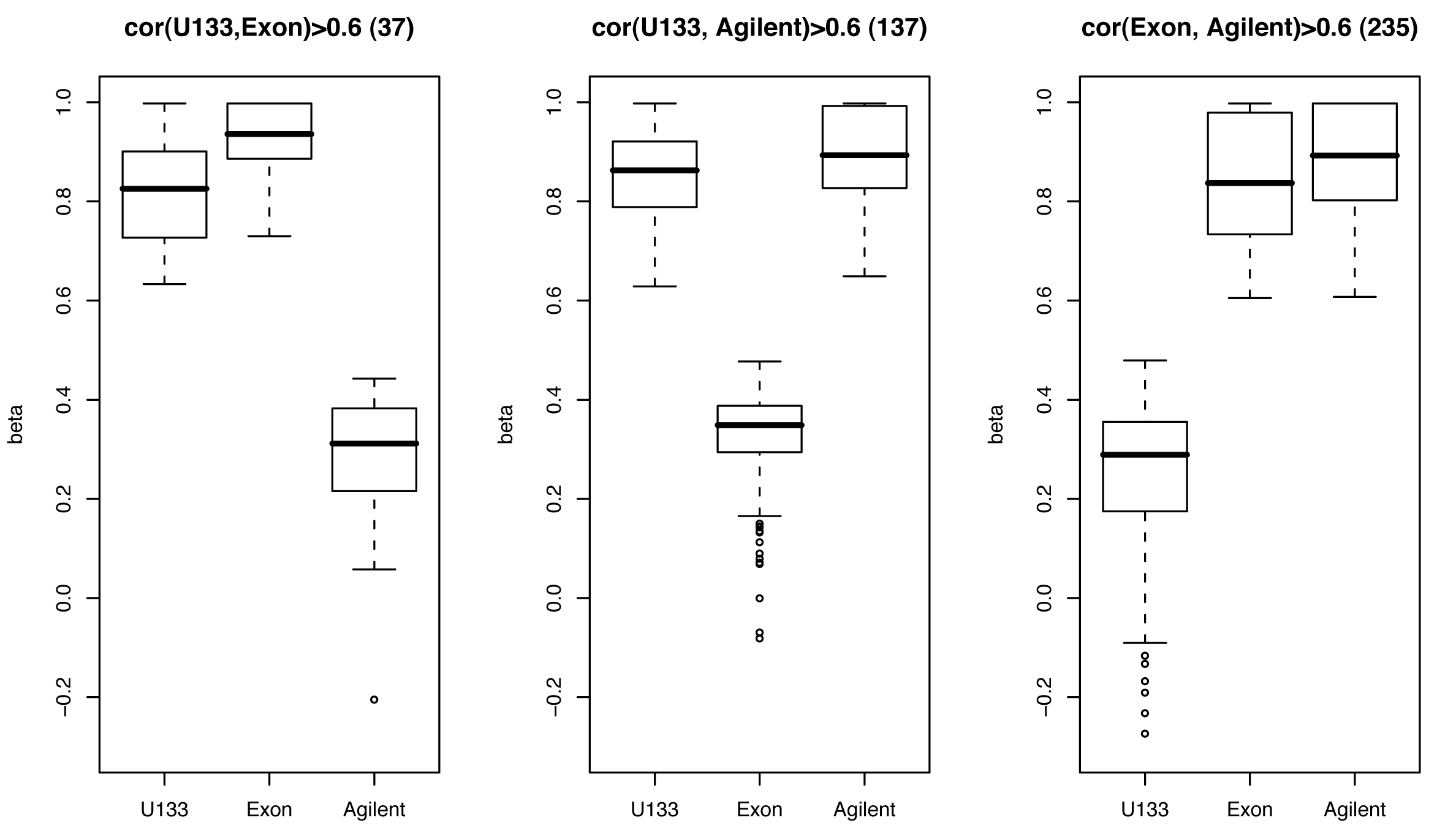

Supplement: Figure S3 — Estimated values for genes where one of the platforms does not correlate with the other two. These are genes where one pairwise correlation between two platforms is and two others are . Gene counts are given in parenthesis in panel titles. (TIF) [file pone.0017691.s003.tif]

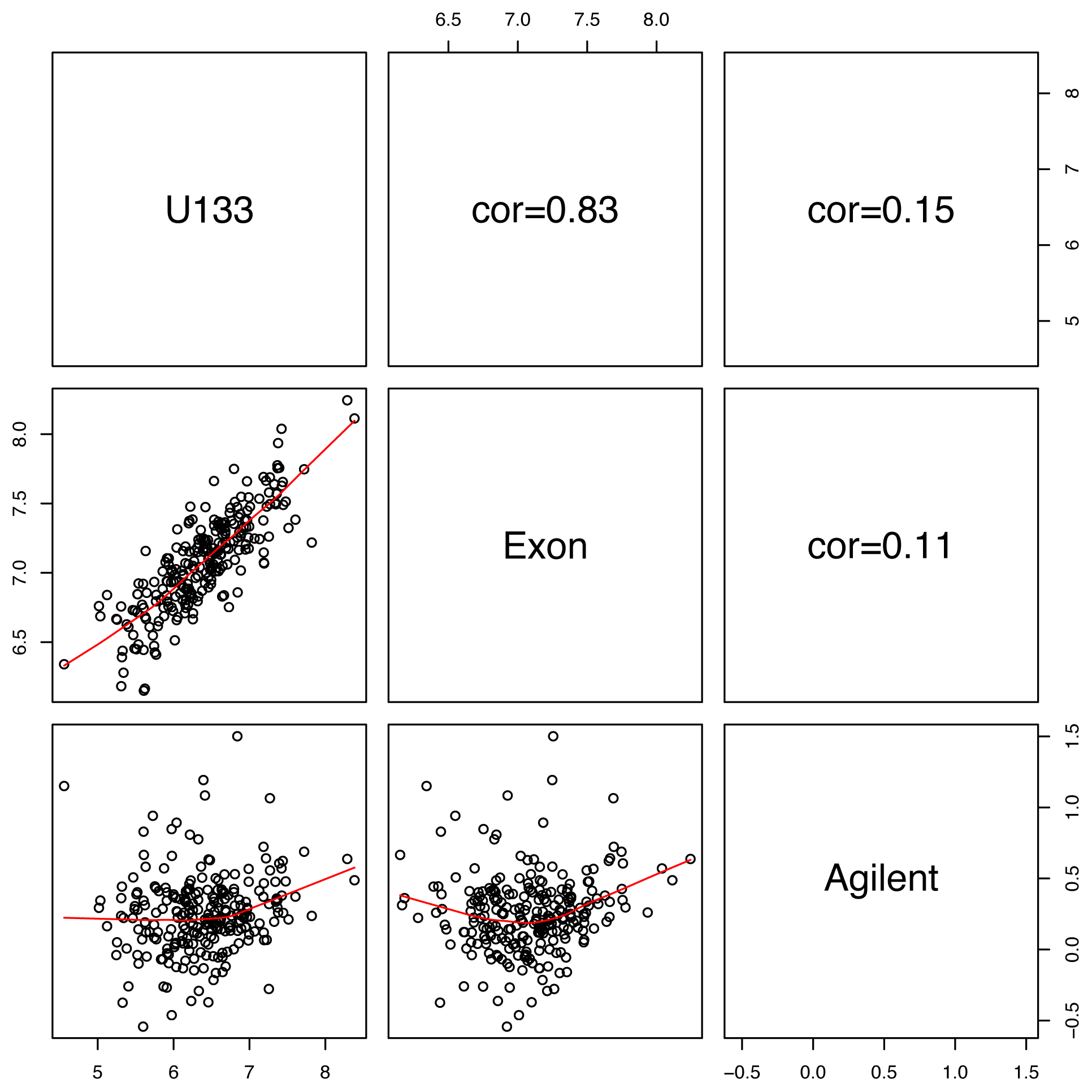

Supplement: Figure S4 — GBM data. Gene SLC36A1: Gene-level summaries. (TIF) [file pone.0017691.s004.tif]

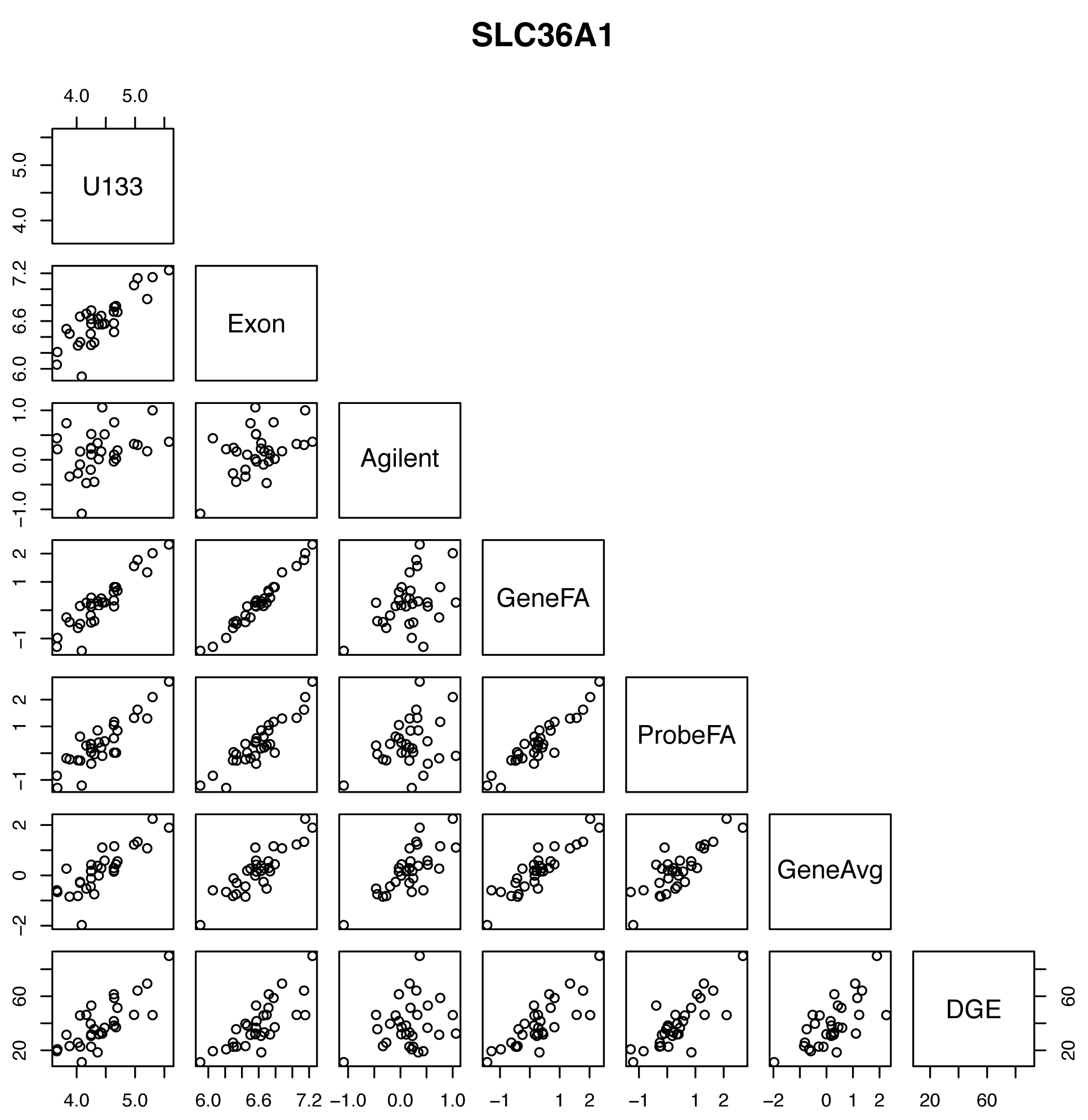

Supplement: Figure S5 — Gene SLC36A1: Gene-level summaries. (TIF) [file pone.0017691.s005.tif]

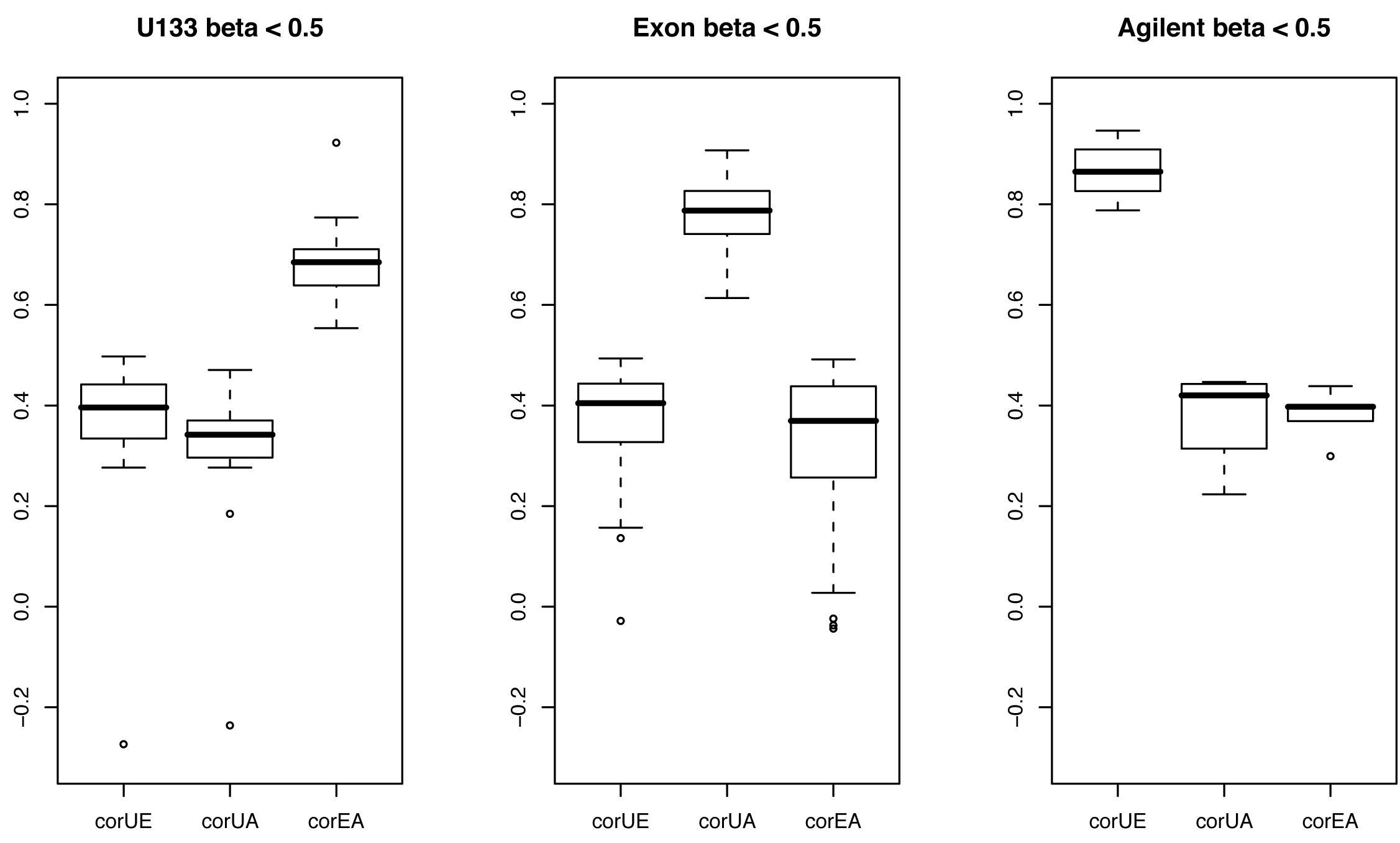

Supplement: Figure S6 — Correlations for 53 genes with one bad value () when the gene is expressed at a reasonable level ( for U133 and Exon, for Agilent) with a reasonable dynamic range (IQR ) on all three platforms, which are likely due to annotation errors. The low value is associated with the platform that is not concordant with the other two. (TIF) [file pone.0017691.s006.tif]

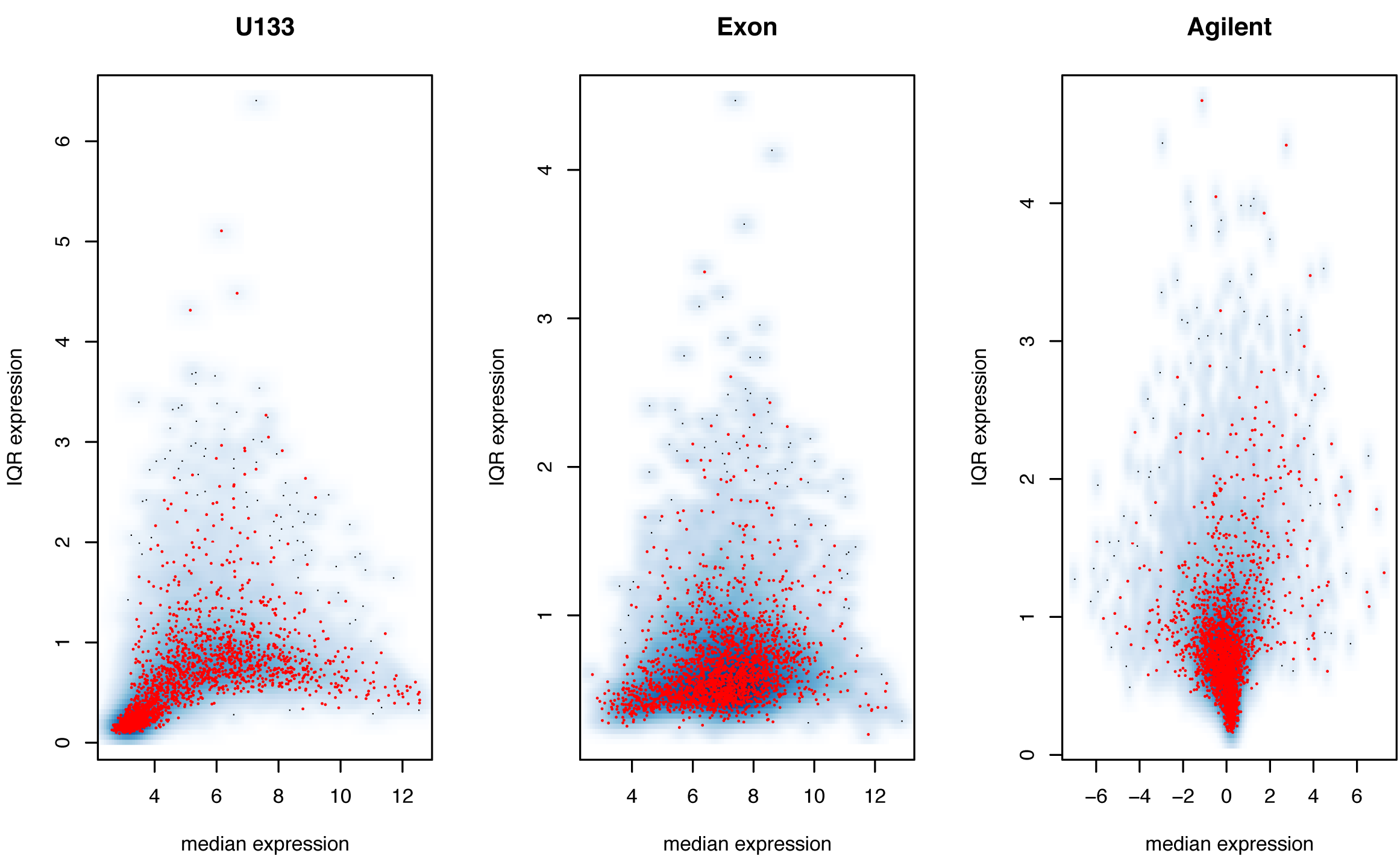

Supplement: Figure S7 — Heywood cases. Red dots are genes in which Heywood cases occur. (TIF) [file pone.0017691.s007.tif]

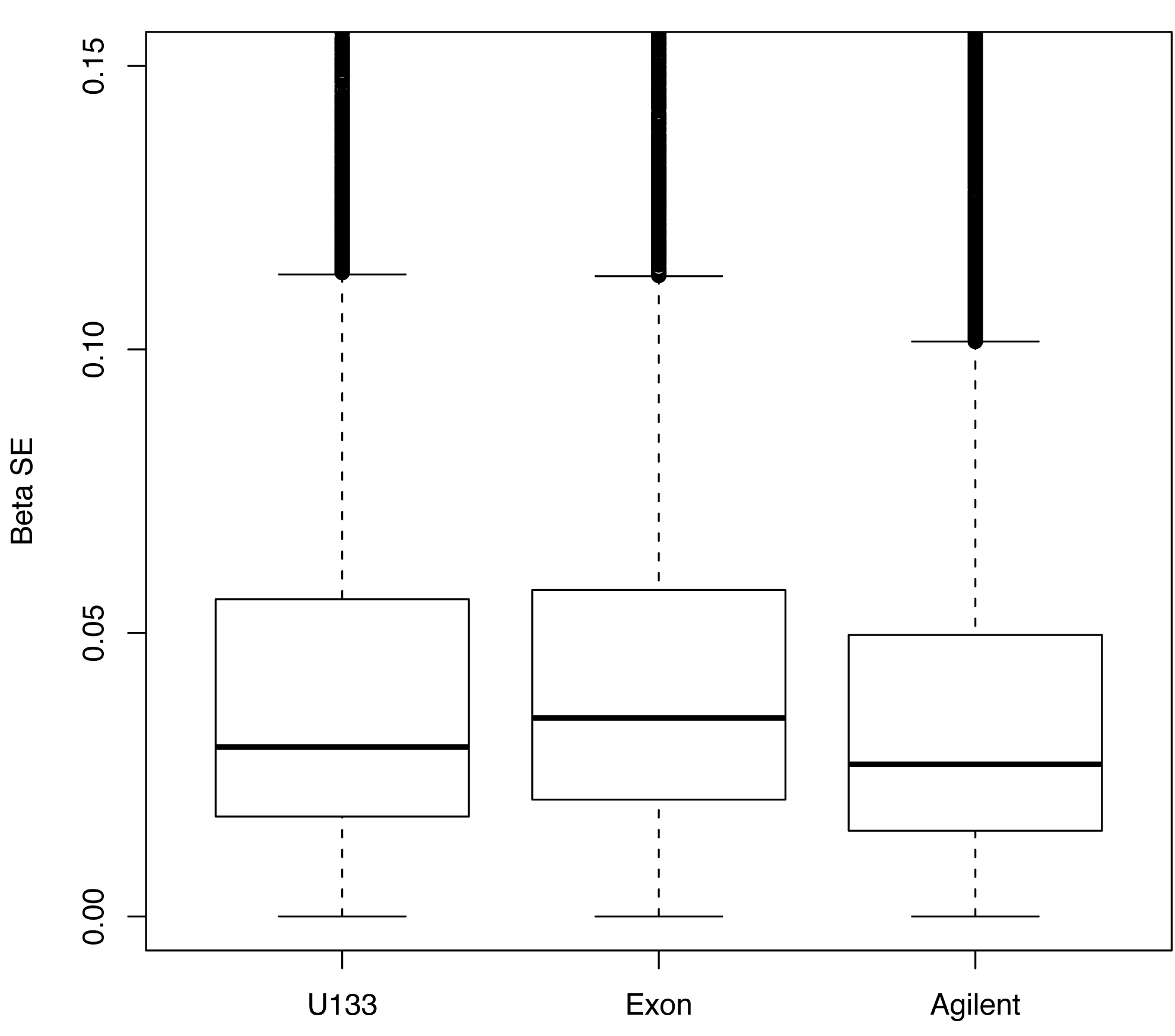

Supplement: Figure S8 — Bootstrap SE of . (TIF) [file pone.0017691.s008.tif]

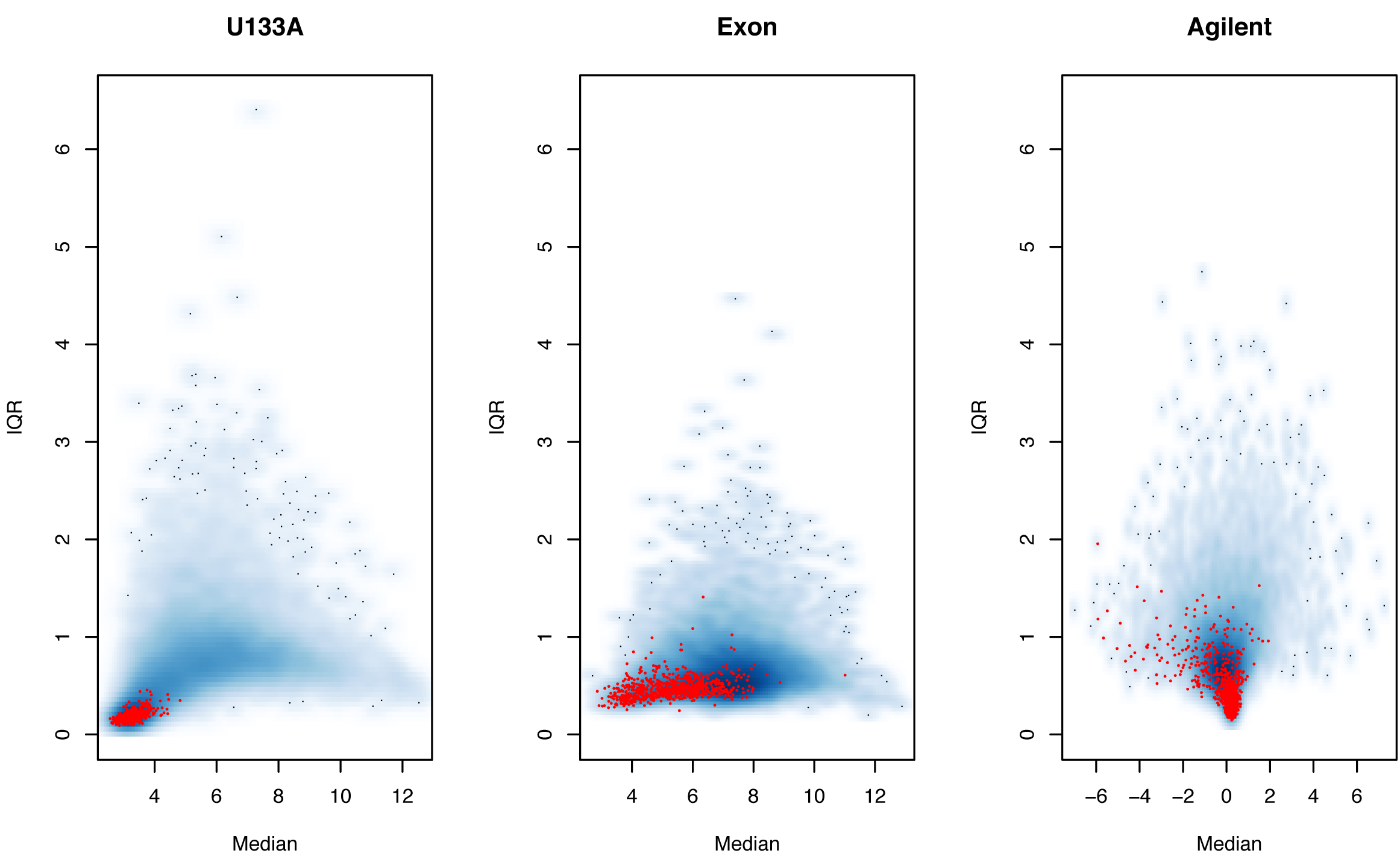

Supplement: Figure S9 — Genes with large bootstrap SE of . Red dots are genes with bootstrap SE of . (TIF) [file pone.0017691.s009.tif]

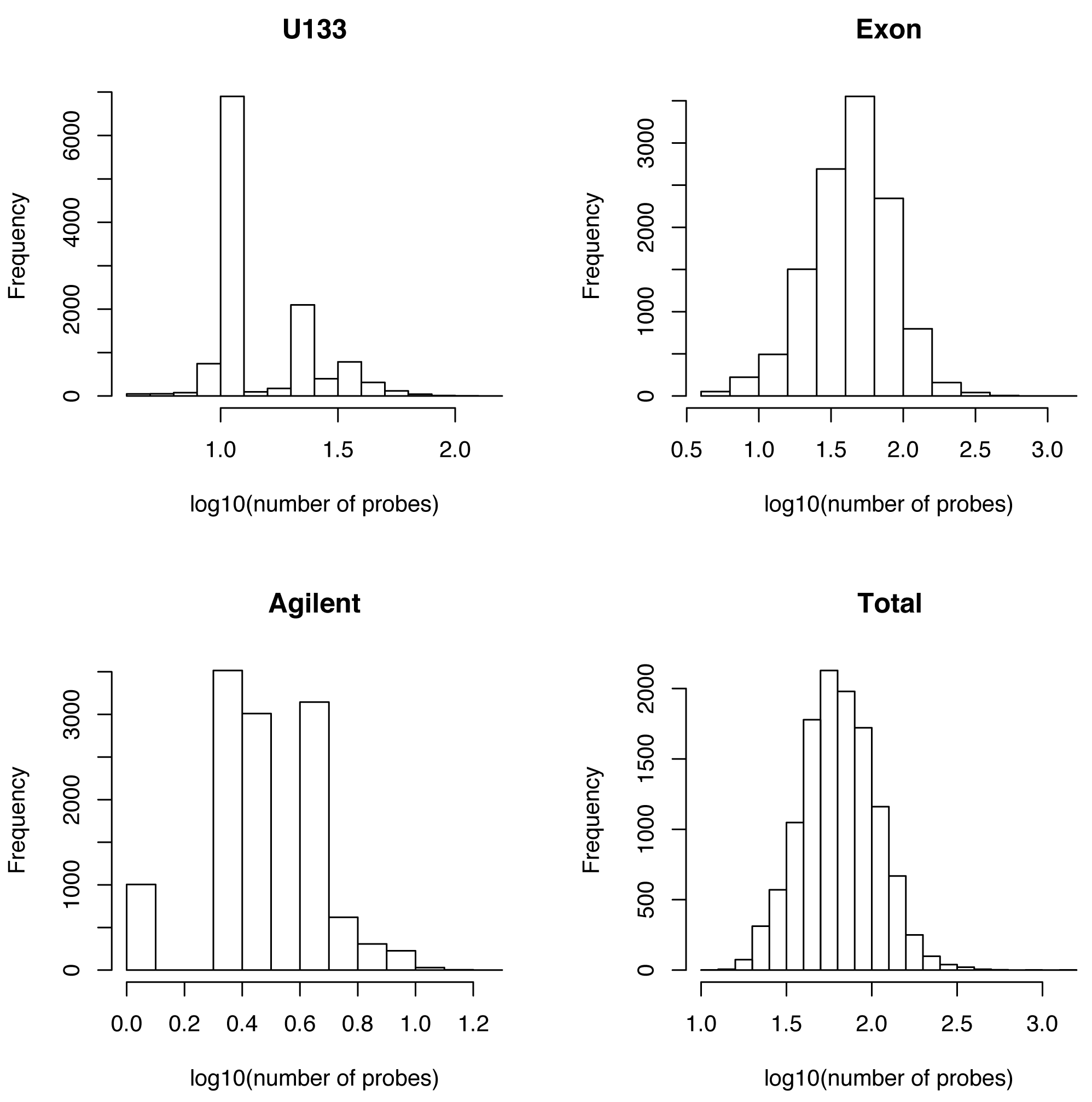

Supplement: Figure S10 — Number of probes per gene on each platform. Figures reflect the number of unique probes on the Agilent array, each usually duplicated 2 to 3 times. (TIF) [file pone.0017691.s010.tif]

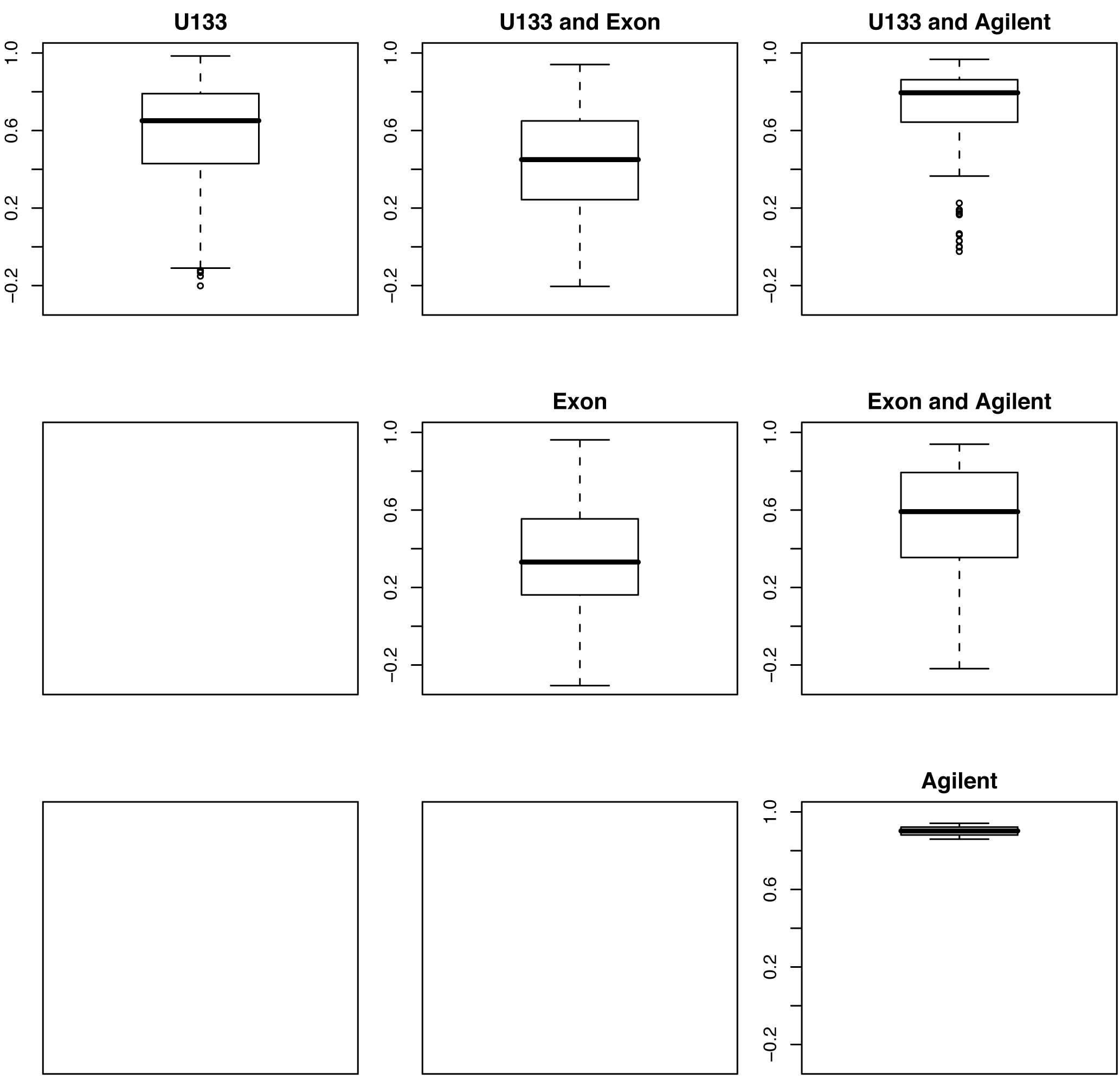

Supplement: Figure S11 — Pairwise probe correlations for gene FHL1 stratified into three within-platform groups and three between-platform groups. FHL1 has 55 U133 probes, 52 exon probes, and 3 Agilent probes. Therefore there are correlations within U133, correlations within exon, correlations within Agilent, correlations between U133 and exon, correlations between U133 and Agilent, and correlations between exon and Agilent. (TIF) [file pone.0017691.s011.tif]

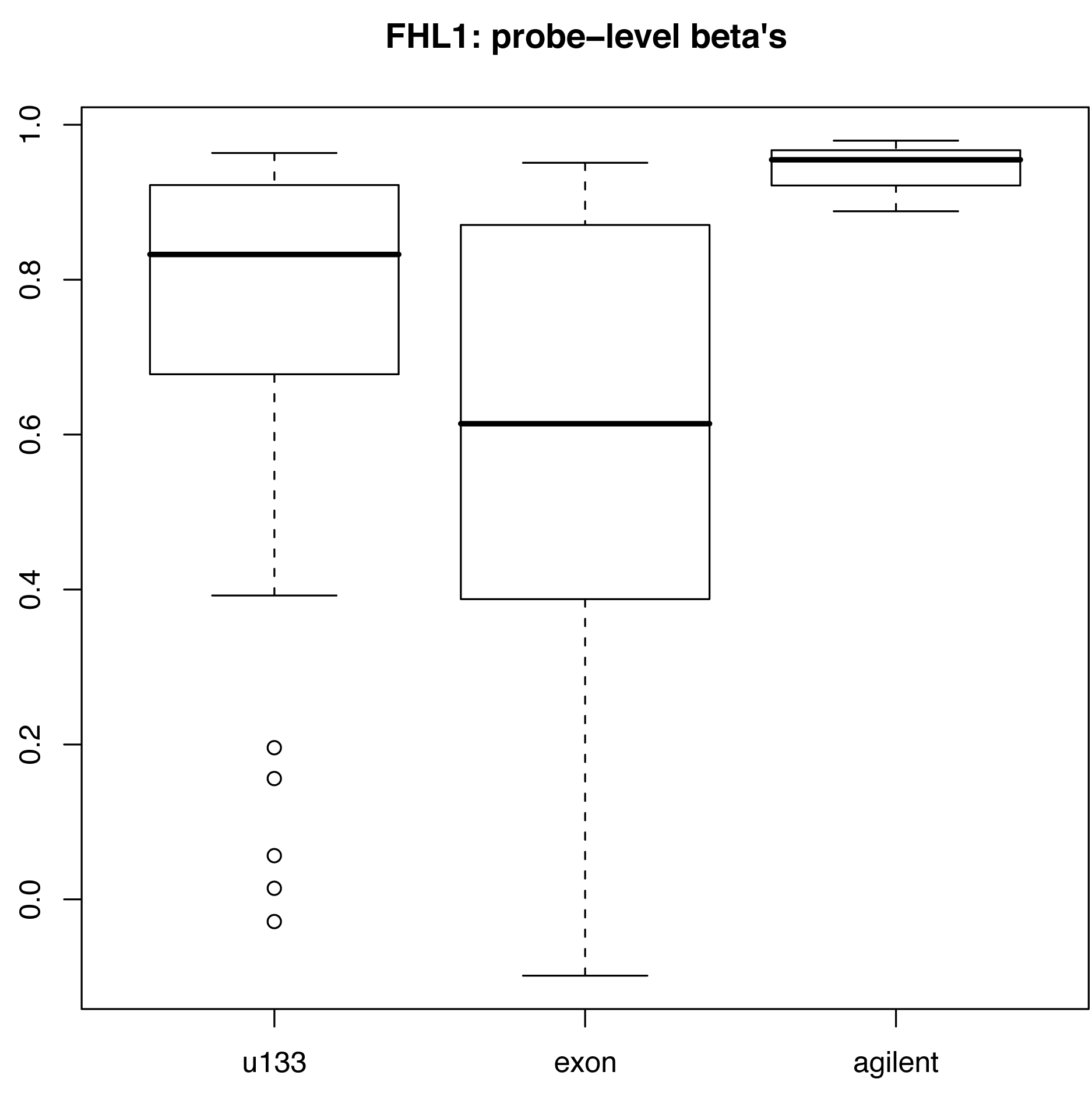

Supplement: Figure S12 — Gene FHL1: probe-level values stratified by platform. (TIF) [file pone.0017691.s012.tif]

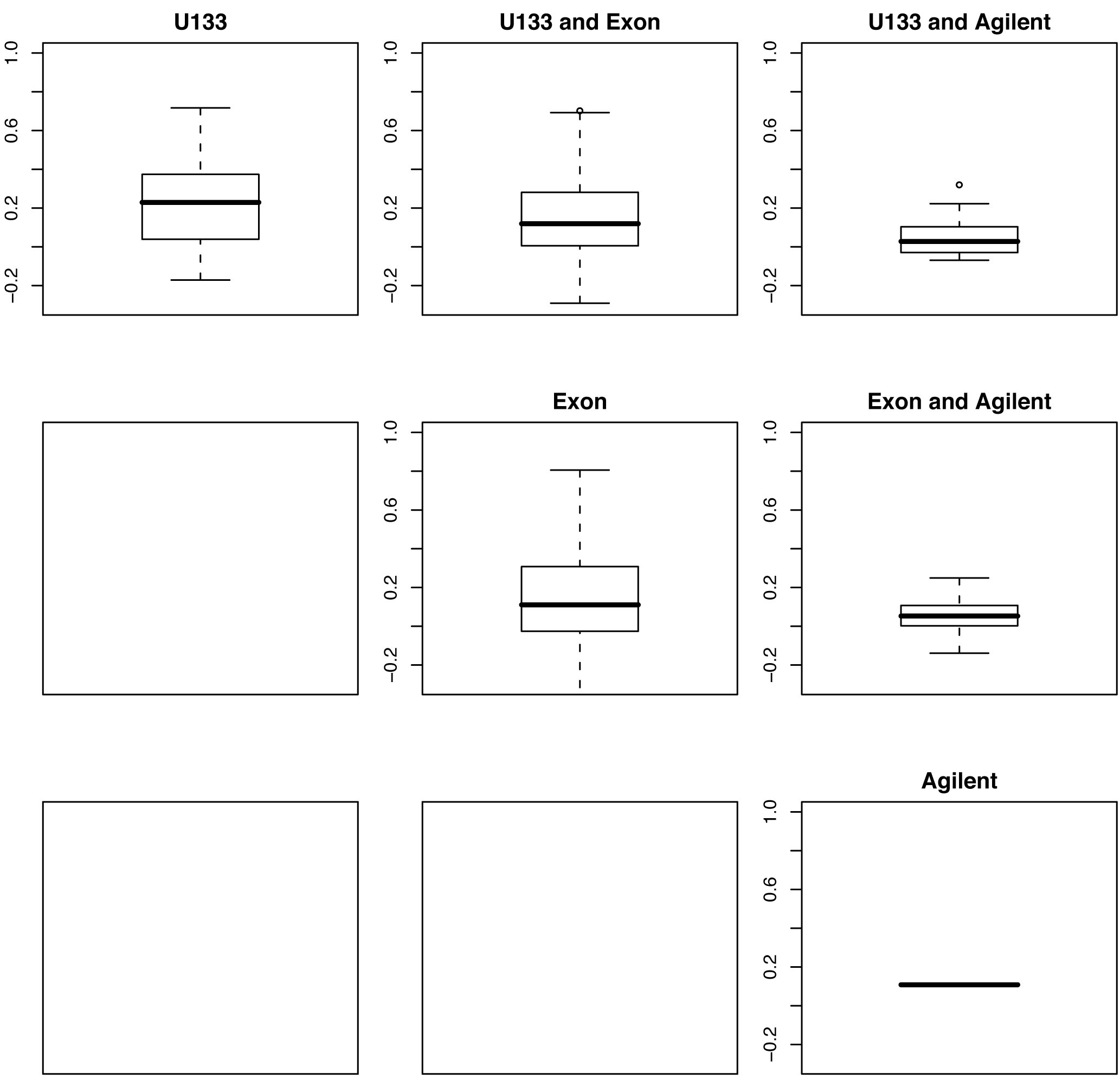

Supplement: Figure S13 — Pairwise probe correlations for gene SLC36A1 stratified into three within-platform groups and three between-platform groups. SLC36A1 has 11 U133 probes, 49 exon probes, and 2 Agilent probes. Therefore there are correlations within U133, correlations within exon, correlation within Agilent, correlations between U133 and exon, correlations between U133 and Agilent, and correlations between exon and Agilent. (TIF) [file pone.0017691.s013.tif]

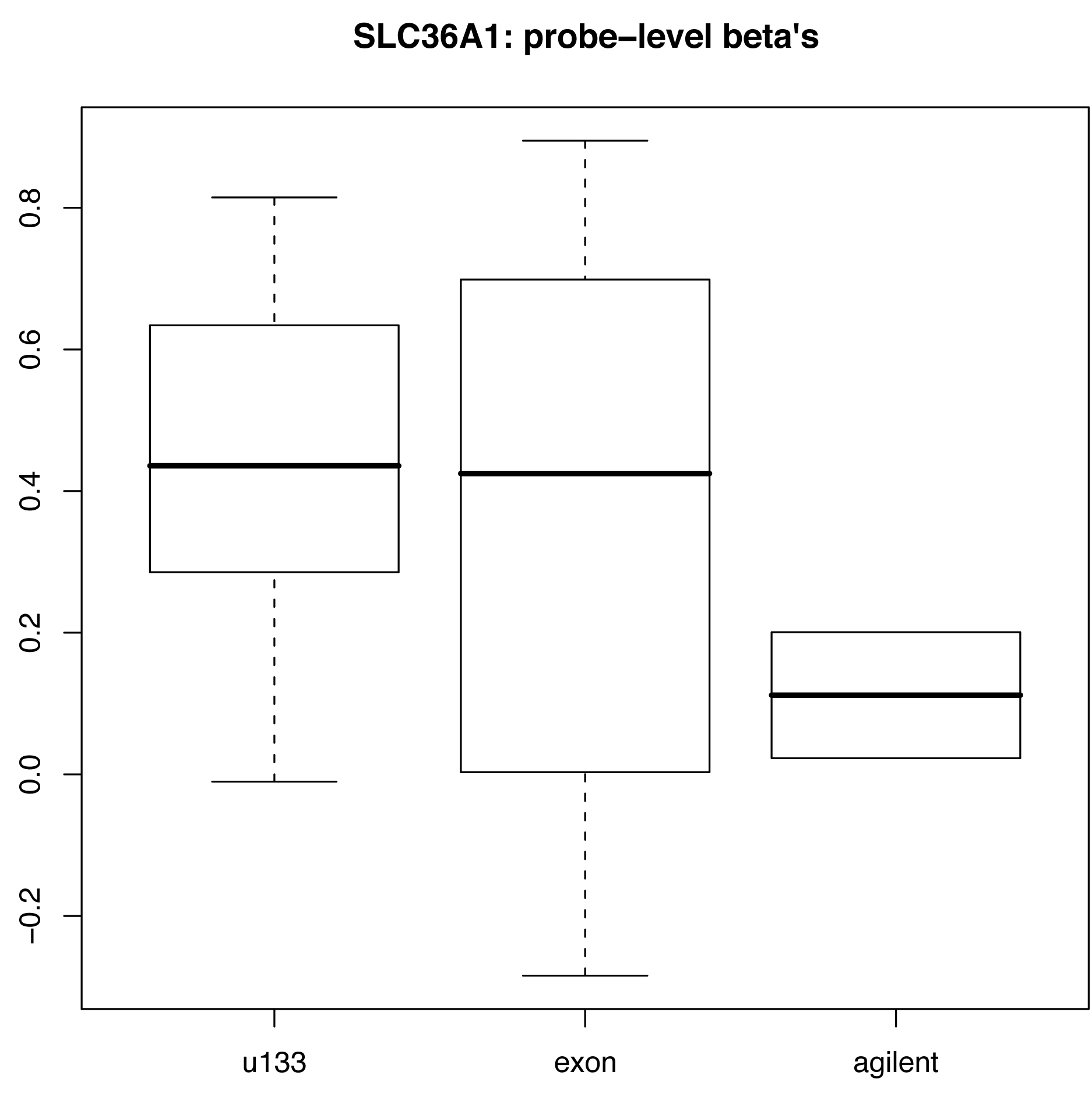

Supplement: Figure S14 — Gene SLC36A1: probe-level values stratified by platform. (TIF) [file pone.0017691.s014.tif]

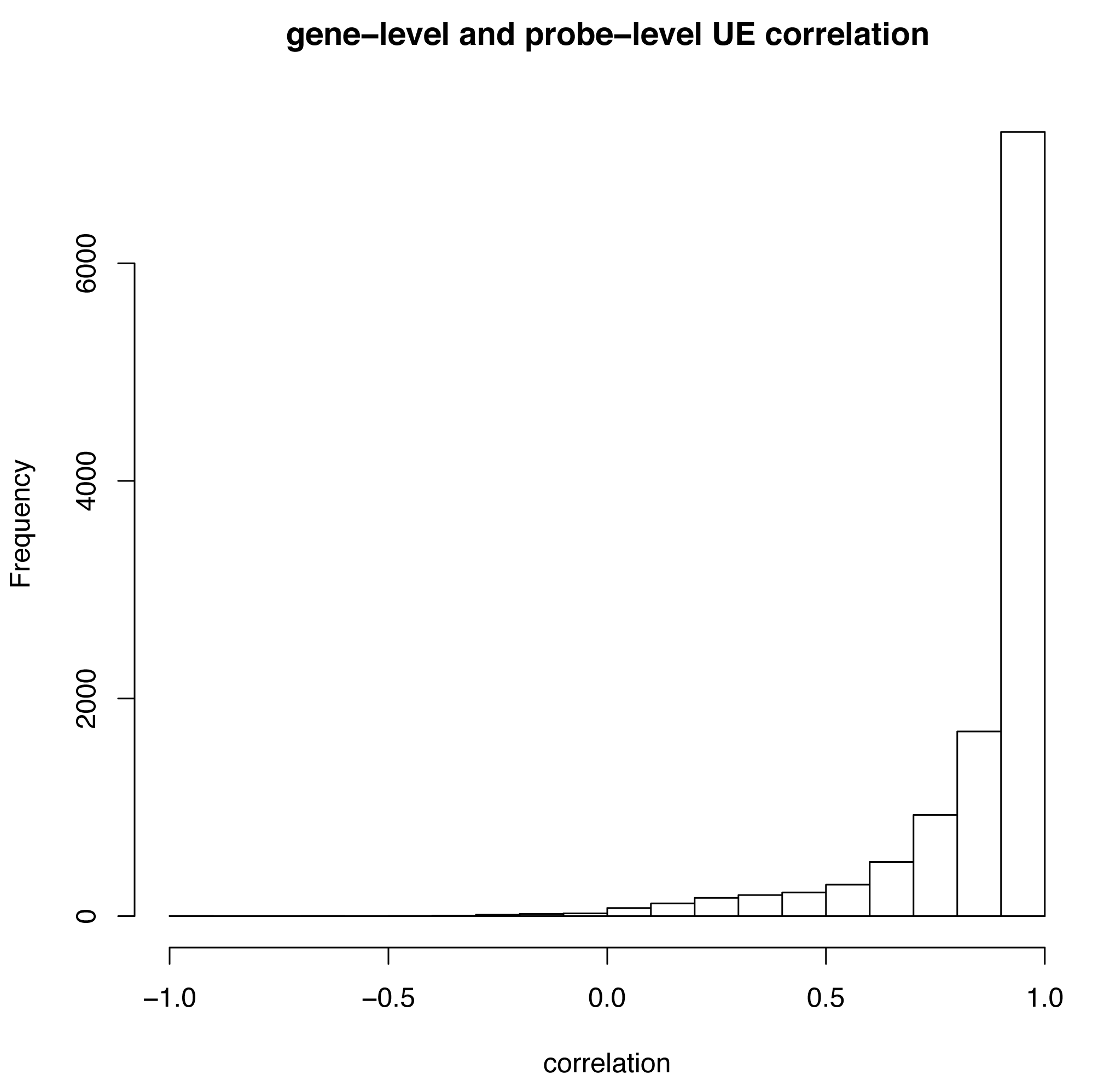

Supplement: Figure S15 — Histogram of 11,864 correlations between matching gene-level and probe-level gene expression estimates. (TIF) [file pone.0017691.s015.tif]

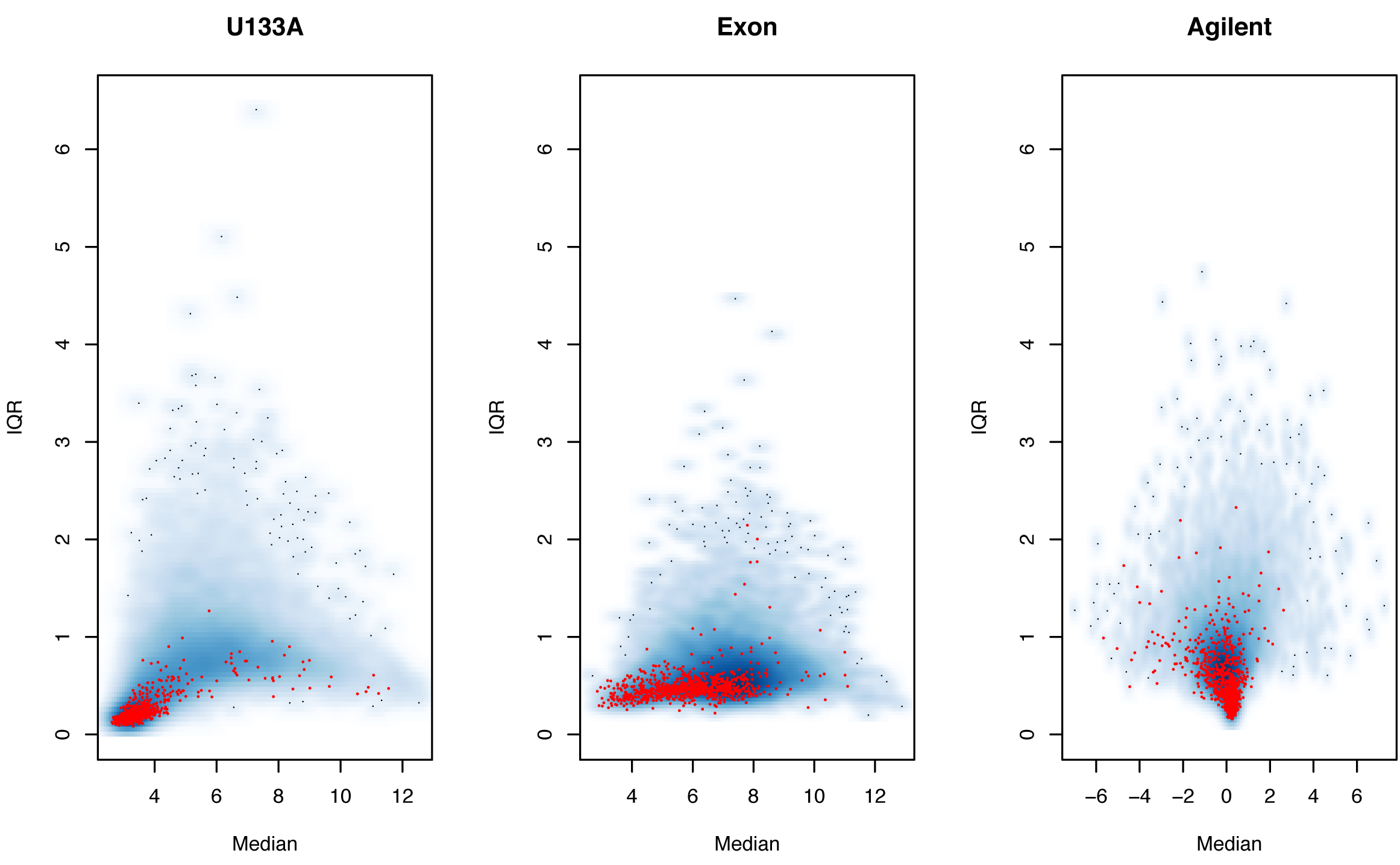

Supplement: Figure S16 — Genes with low correlation between probe-level FA and gene-level FA. Red dots are genes whose correlation between probe-level FA and gene-level FA estimates are below . (TIF) [file pone.0017691.s016.tif]

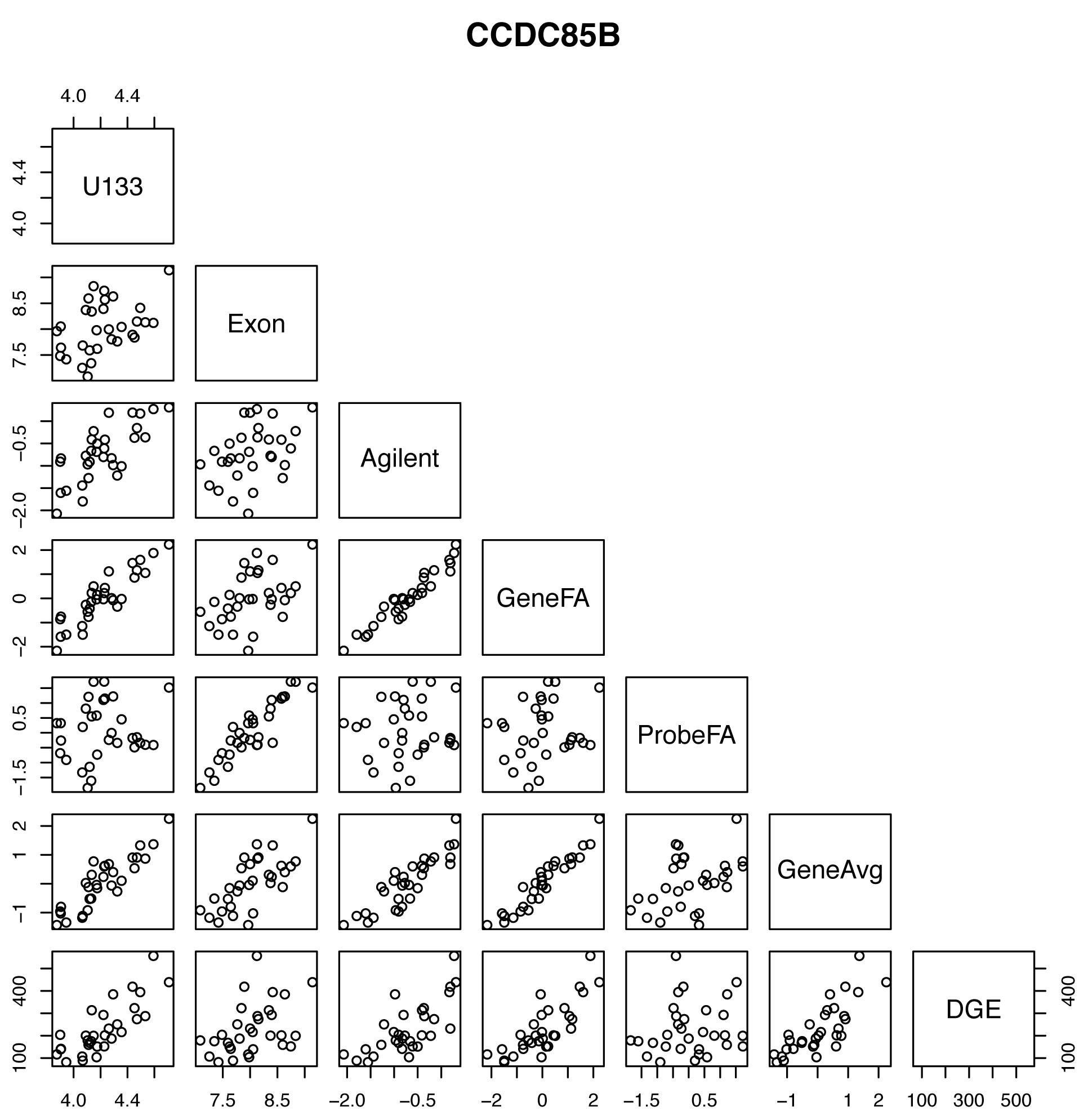

Supplement: Figure S17 — Gene CCD85B. Probe-level FA strongly influenced by Exon array because of its large number of probes. (TIF) [file pone.0017691.s017.tif]

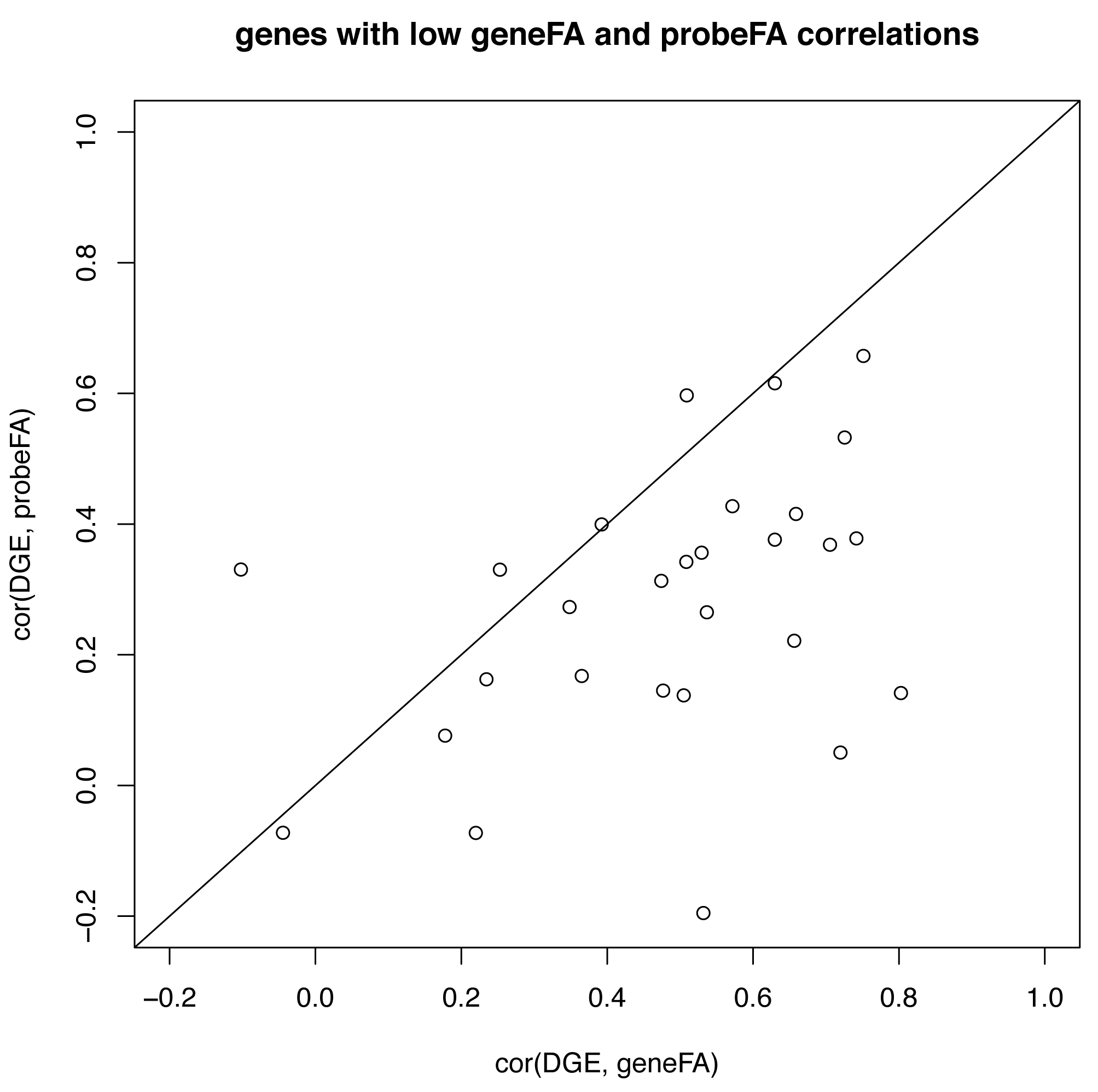

Supplement: Figure S18 — Correlations between gene-level FA and DGE, and correlations between probe-level FA and DGE for 30 genes with low probe-level and gene-level FA correlations. These genes have reasonable expression levels and dynamic ranges, and are not Heywood cases. (TIF) [file pone.0017691.s018.tif]

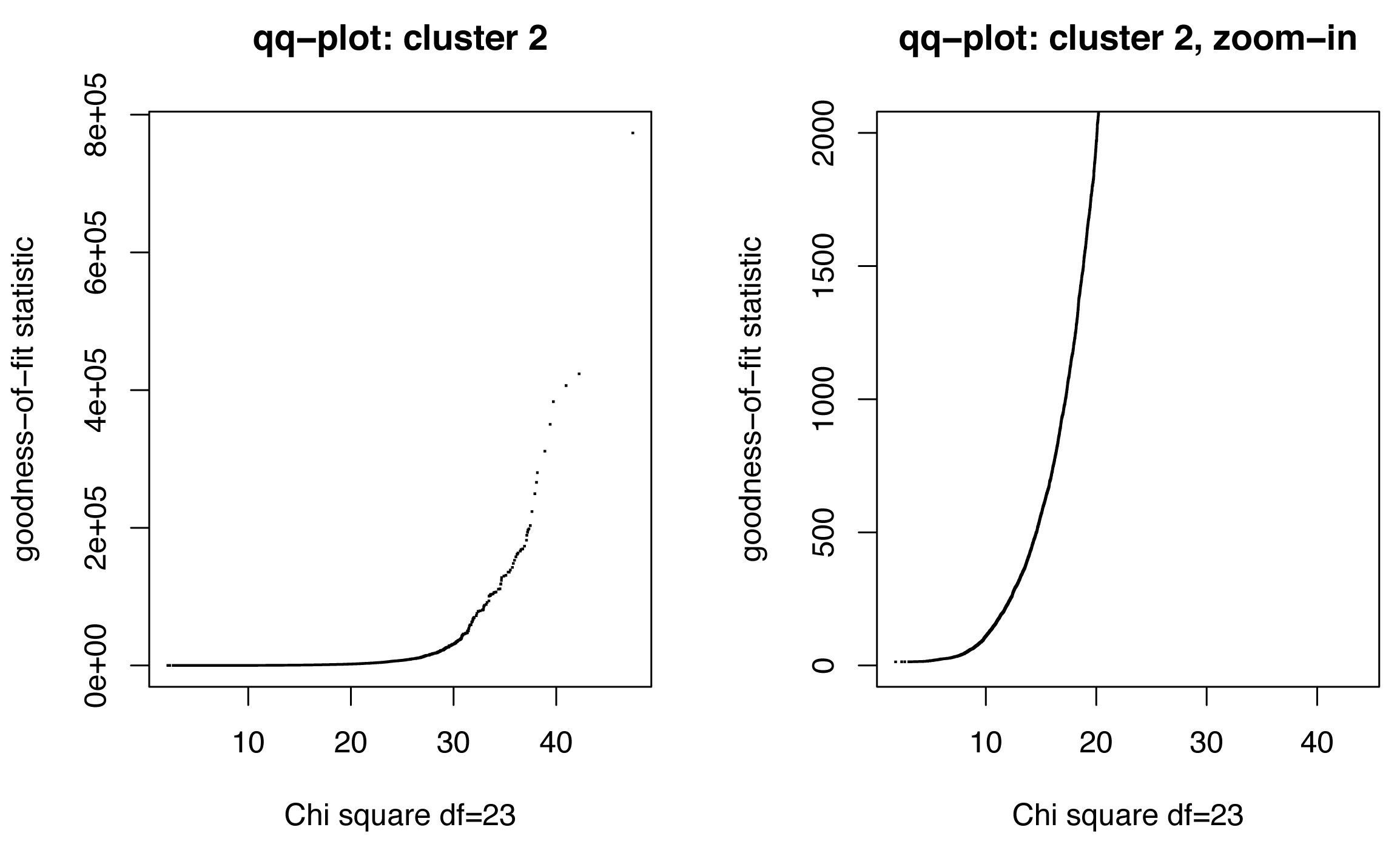

Supplement: Figure S20 — QQ-plot of goodness-of-fit statistics for 24 samples from patients in cluster 2. Under the assumption of homogeneous Poisson rate for the gene counts, the goodness-of-fit statistics is with 23 degrees of freedom. We see significant deviations from homogeneous Poisson distribution. (TIF) [file pone.0017691.s020.tif]
