# Supplementary material for: Unifying Gene Expression Measures from Multiple Platforms Using Factor Analysis
Source: PLoS One. 2011 Mar 11;6(3):e17691. doi: 10.1371/journal.pone.0017691 (PMC3059153; doi:10.1371/journal.pone.0017691)
Supplement: Table S1 — Number of genes with varying cutoffs for all three platforms. (PDF) [file pone.0017691.s021.pdf]

Table S1. Number of genes with varying β cutoffs for all three platforms.

β	number	percentage
> 0	11611	97.9
> 0.1	11404	96.1
> 0.3	10577	89.1
> 0.5	9442	79.6
> 0.7	7193	60.6
> 0.9	1509	12.7
> 0.95	286	2.4
> 0.96	158	1.3
> 0.97	58	0.5
> 0.98	9	0.1
> 0.99	0	0.0
