# Supplementary material for: Unifying Gene Expression Measures from Multiple Platforms Using Factor Analysis
Source: PLoS One. 2011 Mar 11;6(3):e17691. doi: 10.1371/journal.pone.0017691 (PMC3059153; doi:10.1371/journal.pone.0017691)
Supplement: Table S2 — Percentage of small 's. (PDF) [file pone.0017691.s022.pdf]

Table S2. Percentage of small β's.

β	U133	Exon	Agilent
< 0	1.3	0.4	0.5
< 0.1	2.5	1.0	1.0
< 0.3	7.5	4.3	3.3
< 0.5	13.8	10.8	7.0
