# Supplementary material for: Unifying Gene Expression Measures from Multiple Platforms Using Factor Analysis
Source: PLoS One. 2011 Mar 11;6(3):e17691. doi: 10.1371/journal.pone.0017691 (PMC3059153; doi:10.1371/journal.pone.0017691)
Supplement: Table S3 — Number of genes with varying cutoffs for all three platforms. (PDF) [file pone.0017691.s023.pdf]

Table S3. Number of genes with varying β cutoffs for all three platforms.

β	number	percentage
< 0.1	6	0.1
< 0.5	129	1.1
< 0.6	312	2.6
< 0.7	539	4.5
