# Supplementary material for: Unifying Gene Expression Measures from Multiple Platforms Using Factor Analysis
Source: PLoS One. 2011 Mar 11;6(3):e17691. doi: 10.1371/journal.pone.0017691 (PMC3059153; doi:10.1371/journal.pone.0017691)
Supplement: File S1 — File describing the processing of DGE data. (PDF) [file pone.0017691.s024.pdf]

Processing of Tag Profiling data

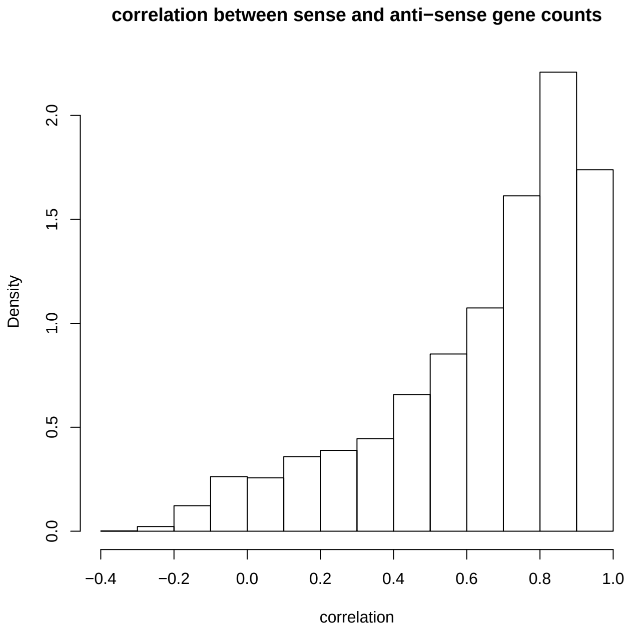

Figure 1: Correlation of gene counts from sense and anti-sense strands. There are a total of 10,207 genes with corresponding unified gene expression measurements in this plot.

We work with processed Illumina Tag Profiling data for 31 of the ovarian tumor samples. A gene can have a number of different tags (median=7), and the total number of times they are sequenced gives the gene expression level. The tags can be on either strand. Usually there are more tags on the sense strand (median=5) than the anti-sense strand (median=2). Figure 1 shows that the counts from the two strands correlate reasonably well for most genes. The genes with low correlations tend to have low expression levels (Figure 2), which is what we would expect.

Therefore we choose to pool the tags from both strands to produce an overall count for each gene. These overall counts are then normalized by the sample median count to correct for different sequencing efforts in different lanes. Let x_{ij} denote the count for gene j of sample i before normalization. Then the normalized count

$$s_{ij} = x_{ij}/\text{median}(x_{i1}, x_{i2}, ...x_{in}) \times C,$$

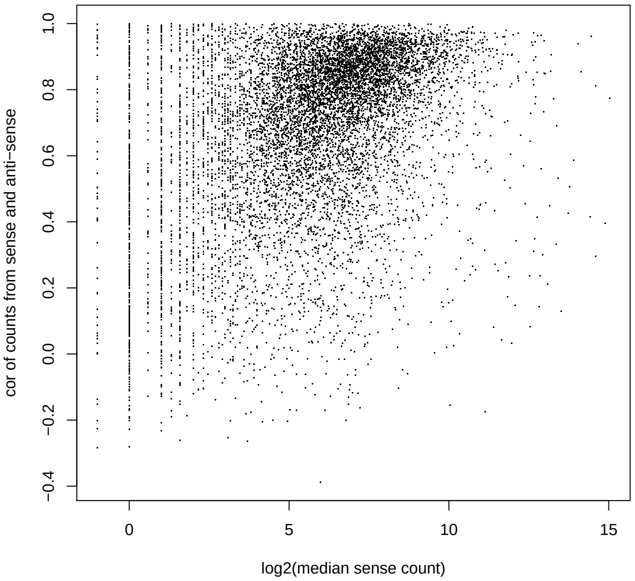

Figure 2: Correlation of gene counts from sense and anti-sense strands by median gene counts from sense strand.

where n is the number of genes and C is a constant chosen so that the normalized data has similar median counts as before. Here we use $C = 50$.

Although Tag Profiling is not the most commonly used technology in obtaining digital measurements of gene expression, 't Hoen et al. (2008) shows that this technology has major improvements in robustness, resolution and inter-lab reproducibility over microarray platforms. It is very similar to the more commonly used Illumina sequencing technology in measuring expression at the gene level. Figure 3 shows the relationship in gene expression measurements between Affymetrix U133 arrays and Tag Profiling for one of the 31 ovarian tumor samples, and it looks very similar to the same figure (Figure 3) in Marioni et al. (2008), which helps with our confidence in this sequencing technology.

References

J. Marioni, C. Mason, S. Mane, M. Stephens, and Y. Gilad. RNA-seq: an assessment of technical reproducibility and comparison with gene expression arrays. *Genome Research*, 18(9):1509, 2008.

P. 't Hoen, Y. Ariyurek, H. Thygesen, E. Vreugdenhil, R. Vossen, R. de Menezes, J. Boer, G. van Ommen, and J. den Dunnen. Deep sequencing-based expression analysis shows major advances in robustness, resolution and inter-lab portability over five microarray platforms. *Nucleic Acids Research*, 2008.

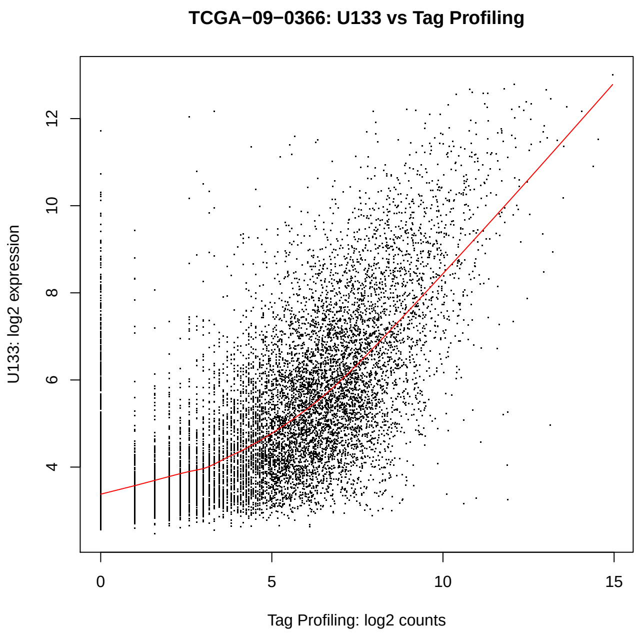

Figure 3: Relationship between gene expression measurements from Affymetrix U133 arrays and Illumina Tag Profiling. We added 1 to Tag Profiling data before taking logs.
